# Catalysts for the Synthesis of Cyclic Carbonates from CO_2_ and Epoxides: A Review

**DOI:** 10.3390/nano16191215

**Published:** 2026-09-26

**Authors:** Weiwei Niu, Shujie Gao, Lemin Zhang, Jinhang Liu, Miying Zhao, Linlin Ma, Lei Wang, Haifeng Ji, Guangwu Li, Husheng Yan

**Affiliations:** 1 Center of Single-Molecule Sciences, Tianjin Key Laboratory of Micro-Scale Optical Information Science and Technology, Institute of Modern Optics, College of Electronic Information and Optical Engineering, Nankai University, 38 Tongyan Road, Jinnan District, Tianjin 300350, China; 1120230185@mail.nankai.edu.cn (W.N.); 1120250258@mail.nankai.edu.cn (L.Z.); 2120230382@mail.nankai.edu.cn (J.L.); 2120240409@mail.nankai.edu.cn (M.Z.); 2120240445@mail.nankai.edu.cn (L.M.); 1120230184@mail.nankai.edu.cn (L.W.); 2 Key Laboratory of Functional Polymer Materials (Ministry of Education), College of Chemistry, Nankai University, Tianjin 300071, China; 3 Beijing Key Laboratory of Lignocellulosic Chemistry, Beijing Forestry University, No. 35, Tsinghua East Road, Haidian District, Beijing 100083, China; msgaosj1999@163.com; 4 Shenzhen Research Institute of Nankai University, 16th Floor, Yantian Science & Technology Building, Haishan Street, Yantian District, Shenzhen 518083, China

**Keywords:** carbon dioxide, cyclic carbonates, epoxides, chemical capture, catalyst

## Abstract

Carbon dioxide (CO_2_) is a major contributor to global warming and a critical concern worldwide. As an economical, abundant, non-toxic, and renewable C1 resource, its utilization is highly significant. The synthesis of cyclic carbonates from CO_2_ and epoxides achieves a 100% atom economy, offering great environmental and economic benefits. Cyclic carbonates are widely used as polar solvents, battery electrolytes, polycarbonate precursors, and chemical feedstocks, showing high application potential. Catalysts lower reaction energy barriers, accelerate reactions, and remain chemically unaltered after the reaction, thus facilitating efficient product isolation. Accordingly, the catalytic conversion of CO_2_ and epoxides has attracted extensive research interest. This review summarizes the catalysts used in this CO_2_ fixation reaction and aims to inspire the development of more efficient catalytic systems.

## 1. Introduction

CO_2_ is a common greenhouse gas and a primary cause of global warming. As the process of industrialization continues to advance, the continuous consumption of fossil fuels such as natural gas, oil, and coal has led to a persistent increase in CO_2_ emissions, which in turn has made the phenomenon of global warming increasingly apparent. This has resulted in a series of hazards including the melting of Antarctic glaciers, rising sea levels, extreme weather events, and species extinction, posing a significant threat to human life, health, and property safety. Therefore, comprehensive global management of CO_2_ is urgently required [1,2,3,4].

At present, the methods adopted by countries in the global management of CO_2_ are still primarily focused on traditional energy conservation and emission reduction, as well as the development and utilization of renewable energy sources. However, it is challenging to significantly reduce the global emissions of CO_2_ in a short period relying solely on conventional technologies. Therefore, Carbon Capture and Utilization (CCU) has emerged as a new approach, transforming CO_2_ into chemical products with higher added value through chemical means. This not only effectively reduces CO_2_ emissions but also turns CO_2_ into a valuable resource, generating additional economic benefits [5,6,7,8,9,10,11]. Utilizing CO_2_ in cyclization reactions to produce high-added-value cyclic compounds is an ideal method of carbon fixation. The compounds formed through cyclization can serve as intermediates in a variety of synthetic reactions and play a significant role in multiple fields, including pharmaceuticals and battery technology. Among these, the reaction of CO_2_ with epoxides to form cyclic carbonates, a process with an atom economy that can reach 100%, has attracted considerable attention from researchers [12,13,14,15]. Cyclic carbonates are chemical products with significant application value in chemical production, possessing numerous excellent properties such as good solubility, biodegradability, and high boiling points. They are widely used in various fields, including non-protic polar solvents, monomers for polymers, raw materials for pharmaceutical production, intermediates in organic reactions, and electrolytes for lithium-ion batteries [16,17,18,19,20,21,22].

However, CO_2_ possesses high thermodynamic stability (with a standard heat of formation of −394 kJ/mol) and kinetic inertness (with a standard Gibbs energy of formation of −395 kJ/mol), which makes the reaction of CO_2_ as a C1-building block with epoxides to form cyclic carbonates a challenge [23,24]. Therefore, to reduce the activation energy for this type of reaction, thereby lowering the required temperature and pressure, various types of catalysts are continuously being developed and applied.

This article presents a review of catalysts utilized in the cyclization of CO_2_ and epoxides to form cyclic carbonates, summarizing and categorizing the various types of catalysts that have been reported in recent years, as summarized in Figure 1. The aim is to provide researchers with a source of inspiration and to offer a reference for the subsequent development of more cost-effective and efficient catalysts.

## 2. Catalyst Systems for the Cycloaddition of CO_2_ with Epoxides

The mechanism for the cyclization of CO_2_ and epoxides to form cyclic carbonates primarily consists of three steps: (1) ring-opening of the epoxide, (2) insertion of CO_2_, and (3) intramolecular cyclization [25,26,27,28,29,30], as shown in Figure 2.

(1) Ring opening of epoxide

Firstly, the Lewis acid (A) or hydrogen bond donor (HBD) interacts with the oxygen atom of the epoxide, leading to the polarization of the C-O-ring bond. Subsequently, the nucleophile (X) attacks the less sterically hindered β-C atom of the activated epoxide, causing the ring-opening of the epoxide and forming an alkoxide intermediate.

(2) Insertion of CO_2_

The negatively charged oxygen atom of the alkoxide intermediate nucleophilically attacks the electrophilic carbon atom of CO_2_, forming an alkyl carbonate intermediate, which results in the insertion of CO_2_.

(3) Intramolecular cyclization

The alkyl carbonate intermediate undergoes intramolecular cyclization, releasing the nucleophile (X) in the process, to form a cyclic carbonate.

The general mechanism for the cyclization reaction that produces cyclic carbonates, as mentioned above, has been distilled through years of relentless exploration by researchers. Building on this foundation, a wider array of catalysts has been discovered. This article provides a comprehensive review of some of the classic types of catalysts that have been reported in recent years, encompassing various categories such as alkali metal salts, ionic liquids, hydrogen bond donors, metal oxides, and more.

### 2.1. Alkali- and Alkaline-Earth-Metal Salt Catalysts

In the catalytic process of converting carbon dioxide and epoxides into cyclic carbonates, alkali metal salts and alkaline earth metal salts fulfill multiple roles: they act not only as Lewis bases to activate CO_2_ but also facilitate the ring-opening reaction of epoxides, stabilize the catalyst structure, and regulate the selectivity of the reaction. Therefore, they play a crucial role in the chemical fixation and transformation of carbon dioxide. As research progresses, the variety of alkali and alkaline earth metal salt catalysts continues to expand.

Building on previous work, Jagtap et al. developed supported liquid-phase catalysts composed of porous silica, polyethylene glycol, and alkali metal halides [31]. Catalytic activity and selectivity strongly depended on the halide type; NaBr performed best, giving 96.39% propylene oxide (PO) conversion at 150 °C and 5.5 MPa CO_2_. In 2010, Wang et al. explored hydrated alkali metal halides [32]. Hydrated salts showed higher activity than anhydrous ones. NaI·2H_2_O exhibited optimal performance, reaching 97% propylene carbonate (PC) yield in 1.5 h at 120 °C and 1.0 MPa CO_2_, with broad substrate applicability.

In 2011, Ghosh et al. studied the synergistic catalysis of alkali metal halides and Lewis bases [33]. Lewis bases greatly enhanced catalytic performance, and the 1:1 molar ratio gave the highest efficiency with up to 91.3% yield. Lithium salts showed better activity than Na/K salts, due to the stronger Lewis acidity and coordination ability of Li^+^. Similarly, in 2013, Shim et al. investigated the cooperative catalytic effect between alkaline-earth metal halides and Lewis bases [34]. Among various magnesium halides and phosphine ligands, MgBr_2_ combined with triphenylphosphine (TPP) achieved the best performance, affording up to 97% yield at room temperature. In 2014, Park et al. demonstrated enhanced catalytic activity by combining natural amino acids with alkali metal halides [35]. The amino acid/KI combination showed much higher activity than either component alone, indicating good synergy between iodide ions and hydrogen-bonding groups in amino acids, which was further supported by density functional theory (DFT) calculations.

In 2015, aiming to develop efficient and recyclable catalytic systems, Werner et al. synthesized polymer-supported catalyst **1** (Figure 3) by combining modified polydibenzo-18-crown-6 with KI [36]. This catalyst showed high activity: at 100 °C under solvent-free conditions, 14 epoxides were converted into cyclic carbonates with yields up to 93%. Two years later, Werner’s group prepared complex **2** in situ from 18-crown-6 and Ca^2+^ [37]. It efficiently catalyzed the conversion of diverse terminal and internal epoxides at room temperature and 1 atm CO_2_, giving extremely high yields and selectivities above 99%.

In 2019, Werner et al. explored alkali/alkaline-earth metal salts combined with polyethers for cyclic carbonate synthesis [38]. CaI_2_ coordinated with poly(ethylene glycol) dimethyl ethers (catalyst **3**) acted as both catalyst and polymeric ligand with excellent activity. It converted 26 terminal epoxides with CO_2_ into cyclic carbonates at room temperature in up to 99% yield. The system also showed high efficiency toward 21 challenging internal epoxides (up to 98% yield) and excellent recyclability.

Similar to Werner’s research, in 2017, Shirakawa et al. also utilized KI for complexation, which combined with tetraethylene glycol to form a catalyst [39]. The results also indicated that the complex formed by the two, complex **4**, exhibits considerable catalytic activity for CO_2_-epoxide cycloaddition. Based on its excellent catalytic performance, in 2020, Butera et al. performed DFT calculations on this KI–tetraethylene glycol system [40]. The results showed that tetraethylene glycol reduced the epoxide ring-opening energy barrier by ~7 kcal/mol, greatly enhancing catalytic activity, and supported the experimental observations.

In 2021, Lyubimov et al. examined the catalytic performance of Na/K halides in CO_2_-epoxide cycloaddition for cyclic carbonates [41]. Only iodides showed activity, while chlorides and bromides were inactive. KI exhibited much higher activity than NaI under the same conditions. This work confirmed the potential of alkali metal iodides and demonstrated that efficient CO_2_ fixation can be achieved by tuning simple inorganic salts, providing important support for designing low-cost and practical CO_2_ conversion catalysts.

### 2.2. Ionic-Liquid Catalysts

Ionic liquids (ILs) are low-temperature molten salts composed of organic cations and inorganic anions, liquids below 100 °C. Some ILs can effectively adsorb and activate CO_2_, which is crucial for cycloaddition reactions. Furthermore, ILs can be immobilized on inorganic materials or polymers to prepare heterogeneous catalysts, maintaining functional–anionic synergies while introducing additional carrier–active site interactions, thus exhibiting excellent catalytic activity and stability. Therefore, ILs have been widely studied for CO_2_ chemical fixation, affording various pyridine-, imidazole-, and ammonium-based functional catalysts.

Previous studies have shown that some imidazolium ionic liquids can act as reaction media and form synergistic systems with metal halides for CO_2_ conversion to cyclic carbonates [42,43]. However, these systems often suffer from low stability, and the use of heavy metals limits their practical application and environmental compatibility. In 2008, Wong et al. designed pyridine-based ionic liquid **5** (Figure 4) [44], which acts as both organocatalyst and reaction medium. It efficiently catalyzed CO_2_ cycloaddition under mild conditions and retained its catalytic activity over multiple cycles, demonstrating good stability and recyclability.

In 2017, Zhang et al. synthesized pyridinium hydrobromide ionic liquid **6,** which showed excellent catalytic efficiency [45]. With only 1 mol% catalyst under solvent-free conditions, various terminal epoxides achieved high conversion and selectivity, and the catalyst could be reused at least five times without obvious activity loss. Its high-performance stems from the cation charge distribution, enabling synergistic activation of epoxides and CO_2_ by acidic protons and bromide ions.

In 2014, based on catalytic synergistic effects, Werner et al. synthesized a series of bifunctional phosphonium salts **7** and first used them as organocatalysts for CO_2_ conversion into cyclic carbonates [46]. Prepared via a simple one-step high-yield synthesis, these catalysts contain hydroxyl side chains. They activate epoxides through hydrogen bonding and electrostatic interactions with the phosphonium center, enabling efficient reaction under mild conditions.

In 2015, Denizalti et al. departed from imidazolium–metal halide synergistic systems and reported a series of β-hydroxyl-functionalized imidazolium salts as highly efficient catalysts [47]. Catalyst **8** exhibited outstanding activity under mild conditions (60 °C, 0.5 MPa, 8 h) and maintained high performance over five reuse cycles. In 2019, Cano et al. synthesized and characterized iron-containing imidazolium ionic liquid catalyst **9**, which showed excellent activity and selectivity [48]. Kinetic and mechanistic studies revealed the active species as [BMIm][Cl] and [Fe(NO)_2_Cl]. [Fe(NO)_2_Cl] acts as a Lewis acid to activate epoxides, while Cl^−^ from [BMIm][Cl] functions as a nucleophile; hydrogen bonding further promotes the reaction.

In 2018, Yang et al. reported a series of multihydroxyl bis(quaternary ammonium) ionic liquids (**10**–**15**) (Figure 5) as metal-free catalysts [49]. They showed high activity and selectivity under solvent- and co-catalyst-free conditions. Under optimal conditions, catalyst **10** gave propylene carbonate with >99% yield and nearly 100% selectivity. In 2019, Liu et al. synthesized novel diionic ammonium salts (**16**–**19**) [50]. Catalyst **18**, with synergistic dual-cation and anion sites, exhibited excellent performance at room temperature.

In 2019, Zhang et al. prepared four amino-functionalized pyrazolium ionic liquids (**20–23**) (Figure 6) for solvent- and co-catalyst-free CO_2_-epoxide cycloaddition [51]. Catalyst **23** performed best, giving 94% PO conversion under mild conditions. In 2024, Wang et al. reported pyrazole-based ionic liquid **24**, which achieved up to 99% yield under mild, metal- and co-catalyst-free conditions with good recyclability [52]. Its poly(ionic liquid) **25** also showed high activity, broad substrate scope, and 99% yield under low temperature and ambient pressure.

In 2025, Ma et al. designed and synthesized a series of protic ionic liquids (PILs) from low-cost amines and acids (Figure 7) [53]. These PILs possess excellent CO_2_ adsorption capacity and high catalytic activity, efficiently catalyzing CO_2_-epoxide cycloaddition under metal- and solvent-free conditions. The system realized efficient conversion under mild conditions (atmospheric pressure, low temperature, short time). Additionally, the PILs exhibited excellent thermal stability up to 373.2 K.

### 2.3. Metal–Salen Complex Catalysts

Metal salen complexes possess characteristics such as CO_2_ activation, intramolecular cooperative catalysis, and ease of recovery and reuse, which can effectively promote the chemical fixation of CO_2_. Based on these properties, various metal salen complexes have been continuously developed and utilized, providing an effective catalytic pathway for the chemical fixation and transformation of CO_2_.

In 2007, North et al. reported a series of bimetallic aluminum(salen) complexes (**26**–**33**) (Figure 8) for the synthesis of cyclic carbonates from CO_2_ and terminal epoxides under ambient pressure and room temperature [54]. When used with tetrabutylammonium bromide (TBAB) as a co-catalyst, these complexes exhibited excellent catalytic activity and high conversion efficiency. In 2011, North et al. further designed single-component bimetallic aluminum(salen) catalysts (**34**–**37**) by covalently immobilizing tetraalkylammonium bromide onto the salen ligand. The resulting catalysts showed good performance toward CO_2_–epoxide cycloaddition [55].

In 2008, He et al. synthesized bifunctional cobalt(salen) complexes (**38**–**45**) (Figure 9) as efficient catalysts [56]. Featuring Lewis acidic metal centers and quaternary ammonium moieties, these complexes showed excellent performance under mild conditions, affording cyclic carbonates with high yield and selectivity without extra solvents or co-catalysts, and could be readily recycled and reused. In 2011, Hong et al. prepared novel bis-urea-functionalized cobalt(salen) complexes (**46–56**) (Figure 10), which self-assembled into structure **57** via intermolecular urea–urea hydrogen bonds. Kinetic and mechanistic studies indicated that the cooperative activation between two cobalt(salen) units induced by such self-assembly contributed to the improved catalytic activity. Intermolecular hydrogen bonding in tetrahydrofuran was further verified by FTIR and ^1^H NMR spectroscopy [57].

In 2021, Hong et al. reported a bimetallic aluminum(salen) catalyst **58** (Figure 11)via hydrogen-bond-mediated self-assembly [58]. The bis-urea-functionalized salen ligand formed a bimetallic framework via urea–urea hydrogen bonds, which greatly enhanced the TOF for cyclic carbonate formation under mild conditions. The TOF was 13-fold higher than that of the monomeric aluminum(salen) catalyst **59** (Figure 12). In 2021, D’Elia et al. synthesized mono- and binuclear Sc/Y-based rare-earth salen complexes (**60**–**64**) and investigated their catalytic performance toward CO_2_–epoxide cycloaddition [59]. These complexes exhibited high catalytic efficiency even at low metal loadings. DFT calculations revealed that, relative to Cr-based salen analogues, Sc and Y complexes possessed lower reaction barriers and reduced steric hindrance, thus accounting for the distinct catalytic activities.

In 2023, Hao et al. constructed three cobalt–salen porous organic catalysts (**65**–**67**) (Figure 13) by integrating cobalt–salen complexes with different acid groups and diaminobenzene-18-crown-6, followed by coordination with KI [60]. Under co-catalyst- and solvent-free conditions, these catalysts converted CO_2_ and various epoxides into cyclic carbonates with yields over 90%. In particular, catalyst **67** showed excellent stability and recyclability, maintaining high activity for at least six cycles.

In 2024, Gu et al. realized the functional integration of cobalt–salen complexes into spirobifluorene-based porous organic polymers via a coordination-immobilization strategy, affording the porous polymeric catalyst ST-CoSalen-POP with covalently bonded Co–salen sites (Figure 14) [61]. This catalyst exhibited a high Co loading (9.35 wt%), large pore volume, and superior CO_2_ adsorption capacity. It efficiently promoted CO_2_–epoxide cycloaddition under mild conditions (25 °C, 0.1 MPa CO_2_), giving cyclic carbonates in up to 96% yield. Moreover, ST-CoSalen-POP showed good substrate generality and excellent recyclability, preserving its structure and activity over three consecutive runs.

### 2.4. Hydrogen-Bond-Donor Catalysts

Hydrogen bond donors (HBDs) are molecules or functional groups that form hydrogen bonds with highly electronegative atoms possessing lone pairs, via their polar hydrogen atoms. In catalysis, HBDs activate epoxides by hydrogen bonding with their oxygen atoms, lowering activation energies for ring-opening reactions and enhancing the efficiency of subsequent cycloadditions. Moreover, HBDs interact with nucleophiles such as halide anions through hydrogen bonding. They stabilize the epoxide oxygen while facilitating nucleophilic attack, thereby promoting epoxide ring opening.

In 2015, Tassaing et al. established a catalytic system composed of cationic salts and various organic hydrogen-bond donors (**68–82**) (Figure 15) for CO_2_–epoxide coupling [62]. Fluorinated donors combined with tetrabutylammonium bromide greatly enhanced the catalytic activity toward diverse epoxides. In 2016, Zhang et al. explored the synergistic effect of tetrabutylammonium iodide (TBAI) and developed a new organocatalytic system using boric acid derivatives (**83**–**109**) (Figure 16) as hydrogen-bond donors. 2,6-Disubstituted arylboronic acids showed superior activity owing to stronger hydrogen-bonding interactions, with water as the optimal solvent. This work highlighted the unique Lewis acid role of boric acid in aqueous media for efficient CO_2_ conversion under mild conditions [63].

In 2017, D’Elia et al. reported ascorbic acid as an eco-friendly and efficient bifunctional hydrogen-bond donor catalyst for CO_2_–epoxide cycloaddition under ambient conditions [64]. Combined with TBAI, it remarkably improved conversion and selectivity. DFT and experimental results clarified the bifunctional role of hydroxyl groups in ascorbic acid during CO_2_ insertion. This work highlights the potential of ascorbic acid as a nontoxic, commercially available, and renewable organocatalyst for CO_2_ fixation, and offers new strategies for designing green catalysts.

In 2021, Guo et al. reported strained ion-pair tris(alkylamine)cyclopropenium halide (TAC·X) catalysts for CO_2_–epoxide cycloaddition under atmospheric pressure, mediated by C−H hydrogen bonding [65]. The positively charged cyclopropenium core converts adjacent C−H/N−H groups into non-classical hydrogen bond donors; NMR titration and computational studies confirmed TAC activates epoxide oxygen via hydrogen bonding, highlighting non-classical C−H donors’ potential for CO_2_ fixation. In the same year, Liu et al. developed a three-component system (pyridine-bridged benzimidazolium salts **110**–**115**, CrCl_2_, TBAB) (Figure 17) for CO_2_–epoxide carbonate synthesis [66]. Under mild conditions (50 °C, 1 bar CO_2_), terminal epoxides showed high reactivity, while internal epoxides were efficiently converted by adjusting reaction parameters. Control experiments and spectroscopy revealed synergistic roles: *N*-heterocyclic carbene precursor activates CO_2_, Cr complex acts as hydrogen bond donor, and TBAB as nucleophile, providing an efficient CO_2_ fixation method.

In 2022, Al-Harrasi et al. reported naturally derived 2-picolinic acid (**116**) as an efficient HBD catalyst for the conversion of CO_2_ with terminal and internal epoxides into cyclic carbonates [67]. The binary system consisting of 2-picolinic acid and *n*-Bu_4_NI afforded yields up to 97% yield and selectivities > 99% at low catalyst loadings (1–2 mol%). Moreover, 2-picolinic acid showed good recyclability, with only a slight loss in activity after five cycles, and scale-up experiments verified the practicality of this catalytic system. In 2024, Guo et al. developed a series of structurally tunable monodentate hydrogen bond donor bifunctional catalysts (**117–131**) (Figure 18) [68]. Systematic studies revealed that iodide-based catalysts with bis-meta-substitution on the aromatic ring showed the best performance. Catalyst **123** converted twelve terminal epoxides into corresponding cyclic carbonates with yields ranging from 72% to 99%.

In 2025, Li et al. synthesized three bidentate HBD catalysts based on deltamides with N–H bonds (**132–134**) (Figure 19), which exhibited good thermal stability [69]. Using SO as a model substrate, the binary system of catalyst **134** bearing a –CF_3_ group and TBAI showed the best performance. Under mild conditions (0.1 MPa CO_2_, 100 °C, 12 h), it achieved 95% conversion and nearly 100% selectivity toward cyclic carbonates. This metal- and solvent-free system offers a green route for cyclic carbonate synthesis and broadens the application of deltamide derivatives in organocatalysis.

### 2.5. Metal–Organic Framework Catalysts

Metal–organic frameworks (MOFs) are crystalline organic–inorganic hybrid materials constructed from metal ions or clusters and organic linkers through coordination bonds. Their high specific surface areas, tunable pore dimensions, accessible metal centers, and chemically adjustable linkers make them attractive heterogeneous catalysts for the cycloaddition of CO_2_ with epoxides. More importantly, their nanoscale pore environments can regulate CO_2_ enrichment, substrate accessibility, the spatial proximity of complementary acid–base sites, and molecular transport. However, excessive confinement may restrict the diffusion of bulky epoxides or retard product desorption. Therefore, the catalytic performance of MOFs should be understood through the combined effects of pore architecture, active-site coordination, local chemical environment, and mass transport rather than surface area alone [70,71,72,73,74,75,76].

In 2013, Kim et al. investigated a series of MOFs with different acid–base properties for the cycloaddition of CO_2_ with styrene oxide [70]. NH_3_- and CO_2_-TPD analyses showed that catalytic activity was more closely associated with the coexistence and distribution of Lewis acidic and basic sites than with surface area alone. The authors proposed that epoxide activation at a Lewis acidic site and CO_2_ adsorption at an adjacent basic site generated a cooperative local reaction environment. Therefore, both the spatial proximity of complementary sites and transport of the reactants to these sites influenced the catalytic performance.

In 2017, Li et al. constructed an acylamide-containing Cu-based MOF with exposed Lewis acidic sites and strong CO_2_ affinity [71]. Small epoxides afforded cyclic carbonate yields of 85–96%, whereas three larger epoxides gave only 6–10%, providing clear evidence that pore accessibility and confinement produced substrate-size selectivity. In 2019, Kim et al. reported that interpenetrated MOF-5, with pore sizes of 7.6 and 6.0 Å, showed approximately twice the activity of conventional MOF-5 despite its smaller pores [72]. DFT calculations indicated that both propylene oxide and styrene oxide could enter the pores and coordinate to Zn(II), illustrating that catalytic performance depends on a balance between confinement, active-site density, and substrate accessibility.

In 2020, Patel et al. reported a dual-ligand CoMOF-2 containing Lewis acidic Co(II) centers and Lewis basic amide functionalities [73]. Because its BET surface area was only 6.8 m^2^ g^−1^, the authors proposed that the reaction occurred predominantly at the external surface rather than within the pore network. DFT calculations supported cooperative participation of the Co centers and ligand functionalities in epoxide activation. This example demonstrates that a nanoscale cooperative environment may also be generated by neighboring surface sites and is not necessarily restricted to internal pores.

In 2021, Gao et al. prepared bimetallic Zn/Mg-MOF-74 catalysts, among which Zn_0.75_Mg_0.25_-MOF-74 exhibited a pore width of approximately 4.5–6.0 Å and the highest CO_2_ uptake and catalytic activity [74]. It afforded a propylene carbonate yield of 99%, whereas a physical mixture of Zn-MOF-74 and Mg-MOF-74 with the same metal ratio gave only 34%, supporting a cooperative effect between adjacent metals within the framework. The lower activity observed for larger epoxides was attributed to restricted intrapore transport, indicating that the internal pores can enrich CO_2_ near acid–base sites but may also limit the accessibility of bulky substrates.

In 2023, Tapiador et al. compared seven Cu-based MOFs and found that the dominant structure–activity factor changed with substrate size [75]. For the relatively small epichlorohydrin, the basicity of the linker functionality was more influential than the total surface area. For the bulkier styrene oxide, Cu-URJC-1 showed the highest activity because its flexible framework and 12 Å channels provided improved substrate accessibility, although the initially slow conversion was attributed to diffusion limitations.

In 2025, Tapiador et al. reported Zn-URJC-12 with two principal pore sizes of 5.3 and 9.9 Å [76]. Under identical conditions, epoxide conversion decreased progressively from propylene oxide to epichlorohydrin, allyl glycidyl ether, and styrene oxide, consistent with increasing steric restriction and hindered pore transport. The modest activity loss during recycling was attributed to partial retention of the cyclic carbonate product through hydrogen-bonding interactions with amino groups inside the cavities. Thus, confinement may enhance reactant activation but can also restrict the diffusion of bulky substrates and retard product desorption.

The performance of MOF catalysts therefore arises from the interplay among pore dimensions, active-site coordination, local acid–base environments, framework flexibility, and molecular transport. Effective MOF design requires sufficient confinement to promote cooperative activation without restricting the accessibility of reactants or the release of cyclic carbonate products [70,71,72,73,74,75,76].

### 2.6. Metal Oxide Catalysts

Metal oxides provide tunable surface acid–base pairs, lattice defects, and multicomponent interfaces for the cooperative activation of CO_2_ and epoxides. In 2018, Tambe and Yadav investigated a La–Zr mixed oxide for the cycloaddition of CO_2_ with styrene oxide [77]. NH_3_- and CO_2_-TPD analyses indicated that weak acidic and strong basic sites participated in epoxide and CO_2_ activation, respectively. The proposed Langmuir–Hinshelwood–Hougen–Watson model further involved adsorption of the two reactants at distinct surface sites. In the same year, Rasal et al. reported a more direct example of defect-controlled catalysis using nanocrystalline Li-doped MgO [78]. Substitution of Mg^2+^ by Li^+^ generated charge-compensating [Li^+^O^−^] defects, described by the authors as F-centers, which promoted CO_2_ activation. However, excessive Li loading caused Li_2_CO_3_ formation, pore blockage, and a decrease in accessible basic sites, demonstrating that defect generation must be balanced against active-site accessibility.

Chowdhury et al. subsequently prepared sheet-like nanocrystalline MgO containing bimodal pores centered at approximately 4 and 18–20 nm [79]. In the presence of TBAB, the catalyst afforded up to 99% cyclic carbonate yield at room temperature and atmospheric CO_2_ pressure. Surface Mg^2+^ and O^2−^ sites were proposed to activate the epoxide and CO_2_, respectively, while the mesoscopic voids provided access to these complementary sites. Kulal et al. further found that Mn–Ba and Sn–Ni mixed oxides were more active than their individual oxides and corresponding physical mixtures [80]. Their catalytic activities correlated with the abundance of weak and moderate acid–base sites, whereas excessive calcination caused particle sintering and loss of accessible sites. These results show that oxide composition and thermal treatment regulate catalytic performance by modifying the coordination and accessibility of surface acid–base sites.

In 2021, Mujmule et al. compared mesoporous ZnCo_2_O_4_ flakes and spheres [81]. The hierarchical flake structure possessed a larger BET surface area and pore diameter than the spherical material and, in combination with KI, achieved 99.9% propylene oxide conversion with at least 99% propylene carbonate selectivity. The authors attributed its performance to the accessible mesoporous architecture and the coexistence of Lewis acidic metal centers and basic surface oxygen sites.

Velpuri and Muralidharan subsequently prepared surfactant-free CuO–ZnO nano-/microflakes with a BET surface area of 45.8 m^2^ g^−1^ [82]. In the presence of TBAB, the mixed oxide afforded a 97% yield for the model reaction, whereas individual CuO and ZnO gave only 35% and 15%, respectively. The authors attributed the enhanced activity to the exposed, uncapped composite surface, although the proposed improvement in electron transfer was not directly demonstrated.

In 2022, Gao et al. established a clearer relationship between solid-solution formation and surface acid–base coordination using Ce_1−x_Zr_x_O_2_ nanorods [83]. Incorporation of Zr distorted and contracted the CeO_2_ lattice, generating additional surface oxygen anions, while Ce_0.7_Zr_0.3_O_2_ possessed the highest density of moderate acidic and basic sites. Epoxide conversion correlated linearly with the amounts of these moderate sites, demonstrating that site strength and coordination were more important than total acid–base-site density. In 2024, Liu et al. compared three MnO_2_ nanostructures and found that δ-MnO_2_, composed of daisy-like nanosheet assemblies, possessed the largest surface area and the highest abundance of moderate acid–base sites [84]. After normalization by surface area, the reaction rate correlated specifically with the amounts of moderate acidic and basic sites, with R^2^ values of 0.997 and 0.992, respectively. Kinetic analysis also revealed an almost zero-order dependence on CO_2_ pressure but a stronger dependence on propylene oxide concentration, consistent with near-saturation of CO_2_ adsorption and a greater kinetic contribution from epoxide activation.

In 2025, Rajalakshmi et al. reported a mesoporous NiO–CuO–ZnO catalyst, NCZ-6, containing particles of approximately 11–50 nm and mesopores of approximately 20–30 nm [85]. In the presence of TPAB, NCZ-6 afforded an 80% cyclic carbonate yield, compared with 23–29% for the individual oxides, supporting a cooperative effect between the acidic and basic sites of the mixed oxide. However, the separate functions proposed for NiO, CuO, and ZnO were not independently verified. Moreover, the yield decreased from 80% to 54% after the first reuse and to 31% by the eleventh run, indicating that long-term stability remained limited despite partial activity recovery after regeneration.

For metal oxide catalysts, the decisive structural parameters are the coordination state of surface atoms, the concentration and nature of lattice defects, and the spatial relationship between acidic and basic sites. Defect engineering and control of oxide composition or interfacial structure can therefore modify CO_2_ and epoxide activation more effectively than simply increasing the geometric surface area [77,78,79,80,81,82,83,84,85].

### 2.7. Porous Organic Polymer Catalysts

Porous organic polymers enable metal centers, ionic groups, hydrogen-bond donors, and CO_2_-philic functionalities to be spatially organized within tunable pore networks. Their catalytic performance is therefore governed by the coupling of pore architecture, active-site accessibility, and the local chemical environment rather than by surface area alone.

In 2016, Alkordi et al. incorporated Cr- and Co-salen complexes into permanently porous organic polymers [86]. Comparison among these materials indicated that the electronic properties and accessibility of the salen sites affected catalytic activity more strongly than the overall surface area. In 2017, Zhong et al. prepared imidazolium–triazine porous polymers containing hydroxyl groups and different counteranions [87]. Anion exchange reduced the BET surface area from 245 m^2^ g^−1^ to 54–67 m^2^ g^−1^ through partial micropore occupation and also changed epoxide conversion from complete conversion to 90% and 31%. These results show that catalytic performance depends jointly on pore accessibility and the local ionic and hydrogen-bonding environment.

In 2018, Wang et al. reported Mg-porphyrin porous polymers containing micropores of approximately 0.5–1.5 nm and mesopores of 3–10 nm [88]. Although increasing the DVB proportion raised the BET surface area from 708 to 2077 m^2^ g^−1^, the turnover frequency decreased from approximately 11,600 to 7000 h^−1^ because the density and accessibility of the embedded Mg sites were reduced. Thus, hierarchical porosity may facilitate molecular transport, but increasing surface area is ineffective when fewer active sites remain accessible. In the same year, Tiffner et al. showed that the activity of cationic ionene polymers was strongly influenced by linker length and counteranion identity [89], further demonstrating that the local ionic environment and halide availability can be more important than bulk textural properties.

In 2020, Ma et al. coordinated Zn(II) to a nitrogen-rich microporous polymer to obtain Zn/HAT-TP [90]. Zn coordination reduced the surface area from 1224 to 396 m^2^ g^−1^ and the CO_2_ uptake from 17.6 to 9.9 wt%, yet markedly enhanced catalytic activity, confirming the central role of accessible Zn coordination sites. In 2021, Guo et al. reported that Co-PPOPs, despite a BET surface area of only 39 m^2^ g^−1^, afforded a cyclic carbonate yield of 98% when combined with TBAB [91]. Control experiments supported cooperation between the Co centers and bromide anions, illustrating how neighboring metal and nucleophilic sites create an effective local reaction environment.

In 2024, Niu et al. developed macroporous polymer beads bearing protonated tertiary amine groups [92]. Although their N_2_-derived BET surface area was only 1.55 m^2^ g^−1^, the iodide-containing protonated material afforded a propylene carbonate yield of 97.6%, compared with 0.62% for the unprotonated polymer, demonstrating the importance of protonation and counteranion reactivity. Liao et al. subsequently prepared mesoporous poly(ionic liquid)s containing imidazolium groups, protonated amine hydrogen-bond donors, and chloride anions [93]. After five cycles, residual cyclic carbonate was associated with a decrease in BET surface area from 226 to 31 m^2^ g^−1^ and an activity loss of approximately 7%, directly showing that product retention and pore occupation can reduce active-site accessibility.

These examples reveal that porosity is beneficial only when the incorporated catalytic groups remain accessible. Micropores can strengthen CO_2_–framework interactions, whereas interconnected mesopores and macropores may improve molecular accessibility; however, site embedding, anion-induced pore blockage, and product retention can offset these advantages. Because quantitative diffusion parameters were generally not reported, the available evidence supports qualitative relationships between pore architecture, molecular transport, and catalytic performance [86,87,88,89,90,91,92,93].

### 2.8. Silica-Supported Catalysts

Mesoporous silica provides an adjustable nanostructured platform for immobilizing organic bases, ionic liquids, metal complexes, and hydrogen-bonding groups. Its catalytic role depends on particle and pore dimensions, the dispersion and accessibility of confined species, and interactions between active components and surface silanol groups rather than on surface area alone.

Barbarini et al. immobilized a guanidine catalyst on MCM-41 with an average pore diameter of approximately 3 nm [94]. Although marked substrate-size limitations were not observed for terminal epoxides, the slower reaction relative to the homogeneous catalyst suggested some diffusion resistance. North and Villuendas subsequently showed that silica-supported aluminium(salen) catalysts based on particles smaller than approximately 80 μm were generally more active, whereas pore size had little effect under the investigated conditions [95]. Moreover, increasing the linker length raised catalyst loading approximately threefold but reduced intrinsic activity by a similar factor, demonstrating that transport distance and the immobilization environment can be more important than pore size or loading alone.

Cheng et al. grafted triazolium ionic liquids onto SBA-15, decreasing the BET surface area from 534.2 to 306.3 m^2^ g^−1^ and the pore diameter from 6.5 to 3.8 nm [96]. Despite partial pore occupation, the functionalized material was substantially more active than bare SBA-15, particularly when hydroxyl or carboxyl groups were present. Similar cooperation between a hydrogen-bond donor and a nucleophilic halide was observed for hydroxy-functionalized ammonium iodide immobilized on silica [97]. These results show that neighboring ionic and hydrogen-bonding sites can create an effective local environment for epoxide activation and ring opening.

Further evidence for the importance of pore accessibility was obtained using imidazole–urea-functionalized mesoporous silica and ionic-liquid-modified SBA-16 [98,99]. Increasing the organic content partially occupied the pores, whereas materials combining open mesoporous channels with accessible hydrogen-bonding, ionic, and nucleophilic sites showed higher activity. Notably, functionalization of SBA-16 reduced its BET surface area but increased CO_2_ adsorption to approximately 3.5 times that of the unmodified support [99], indicating that the local chemical environment can outweigh the loss of geometric surface area.

A more explicit confinement effect was demonstrated by Su et al. for EmimBr assembled within mesoporous silica [100]. The material containing 6.9 wt% EmimBr exhibited the highest ionic-liquid dispersion and a turnover frequency approximately 1.7 times that of bulk EmimBr at an equivalent active-site amount. XPS analysis and DFT calculations indicated that exposed silanol groups strengthened the interaction between the imidazolium cation and bromide anion. Thus, confinement improved active-site dispersion and accessibility while modifying and stabilizing the local ionic environment.

More recent studies extended these concepts to inorganic and organometallic active sites. An amorphous mesoporous titanium–silica material containing polydiallyldimethylammonium chloride (PDDA) within 10–20 nm pores combined basic sites for CO_2_ activation with Lewis acidic Ti sites for epoxide activation [101]. Its higher activity than crystalline Ti-mordenite was partly attributed to the greater accessibility of sites in the loose mesoporous structure. Ag nanoparticles supported on sponge-like TUD-1 silica likewise generated metallic Ag and interfacial Ag–O–Si species [102]. Although a direct nanoparticle-size–activity relationship was not established, this system illustrates how mesoporous silica can stabilize nanoscale metal species and create distinct metal–support interfaces.

Kracht et al. subsequently grafted Mg, Al, and Ti pyrazolate complexes onto SBA-15, producing site-isolated monometallic surface species while retaining mesoporosity [103]. The Mg-containing hybrid was more active than its homogeneous analogue, including for bulky epoxides. This enhancement was attributed to surface confinement, site isolation, modified coordination geometry, and the electronic influence of the silica surface. A molecular Ti model also formed a carbamate species upon CO_2_ insertion, supporting the participation of the grafted coordination environment in CO_2_ activation.

The available evidence therefore points to a coupled effect of particle size, mesopore accessibility, active-site dispersion, and interfacial coordination or hydrogen-bonding interactions. Confinement can isolate catalytic species and generate cooperative environments with surface silanols, whereas excessive functionalization or unfavorable immobilization may block pores and restrict site accessibility. Because most studies did not report quantitative diffusion parameters, these observations establish qualitative structure–transport relationships rather than definitive diffusion kinetics [94,95,96,97,98,99,100,101,102,103].

### 2.9. Carbon-Based Catalysts

Carbon-based catalysts offer tunable defect density, heteroatom composition, pore structure, and atomic coordination environments. Their catalytic behavior is therefore determined by the chemical identity and accessibility of defect or dopant sites, rather than by surface area alone.

Su et al. showed that decreasing the preparation temperature of urea-derived g-C_3_N_4_ reduced its crystallinity and polymerization degree, creating more edge defects containing incompletely coordinated nitrogen atoms [104]. Catalytic activity followed the concentration of these defect-derived sites, identifying undercoordinated nitrogen as a major contributor to CO_2_ activation. Huang et al. further demonstrated that acid treatment fragmented g-C_3_N_4_ into 20–50 nm particles and generated structural defects, terminal amino groups, and hydroxyl groups [105]. A high-surface-area control remained much less active, indicating that the enhanced activity arose mainly from the defect-derived chemical environment rather than from surface area alone. The authors proposed that amino groups activate CO_2_ while hydroxyl groups polarize the epoxide through hydrogen bonding.

Nitrogen-doped porous carbon nanofiber webs similarly combined adjustable pore structures with nitrogen functionalities for CO_2_ capture and conversion [106]. However, subsequent biochar studies provided clearer evidence that surface chemistry could dominate textural properties. Vidal et al. found that oxidized hardwood and softwood biochars exhibited comparable activity despite markedly different surface areas [107]. Blocking their surface carboxylic acid groups eliminated catalytic activity, supporting hydrogen-bond activation of the epoxide by these groups.

Heteroatom co-doping enables complementary sites to be positioned within the same carbon framework. Ghosh et al. prepared hollow-sphere N/S-co-doped carbon composed of interconnected nanosheets [108]. Experimental results and DFT calculations indicated preferential CO_2_ adsorption at pyridinic nitrogen and epoxide adsorption at terminal–SO_2_H sites, producing a cooperative acid–base microenvironment. Cheng et al. reported a related N/O-co-doped porous biochar in which nitrogen- and oxygen-containing groups acted as complementary Lewis basic and acidic sites [109]. Theoretical calculations supported simultaneous adsorption and activation of CO_2_ and the epoxide. Although the hollow and porous architectures provide accessible surfaces, neither study quantified intraparticle diffusion; their strongest evidence concerns spatially integrated acid–base functionality.

More defined coordination environments were achieved by anchoring metal atoms to nitrogen-doped carbon. Huang et al. prepared a Zn-supported nitrogen-rich carbon in which Zn species were uniformly dispersed and stabilized by nitrogen sites [110]. Raising the calcination temperature promoted Zn aggregation and crystallization while decreasing the contents of pyridinic and pyridonic nitrogen, leading to lower catalytic activity. This inverse relationship shows that atomic dispersion and the local Zn–N environment are more important than the modest increase in surface area at higher calcination temperatures.

Yang et al. constructed porous carbon nanosheets containing approximately 14.4 wt% atomically dispersed Al in Al–N_4_ coordination environments [13]. Aberration-corrected HAADF-STEM and X-ray absorption spectroscopy confirmed isolated Al atoms and planar Al–N_4_ motifs. In this structure, Al and neighboring N sites functioned as Lewis acidic and basic centers, respectively. Experimental and theoretical analyses further indicated that light irradiation promoted electron transfer from Al–N–C to the epoxide, facilitating the rate-limiting ring-opening step. This system provides direct evidence that atomic coordination and the electronic structure of isolated sites can govern catalytic activation.

Zhang et al. extended the cooperative-site strategy to B-, N-, and P-containing porous carbon [111]. BPO_4_@BCN-800 contained both micropores and mesopores together with Lewis acidic B species, Lewis basic N species, and Brønsted acidic functionalities. The authors proposed that these neighboring sites cooperatively enriched CO_2_, polarized the epoxide, and promoted ring opening. Because the catalytic mechanism was inferred from characterization and control experiments rather than direct observation of all intermediates, it should be regarded as a supported mechanistic proposal.

The carbon-based systems discussed above demonstrate that defect density, heteroatom distribution, atomic coordination, and pore accessibility must be considered together. Porous or hollow structures can expose catalytic functionalities, but the decisive factor is whether undercoordinated nitrogen, hydrogen-bond donors, complementary heteroatoms, or isolated metal–N sites form accessible local environments. Claims regarding enhanced diffusion should remain qualitative because direct transport measurements were generally not reported [13,104,105,106,107,108,109,110,111].

To facilitate a comprehensive comparison of the catalytic systems discussed above, Table 1 summarizes representative catalysts reported for the cycloaddition of CO_2_ with epoxides, along with their reaction conditions and catalytic performance. Key parameters, including catalyst type, substrate, CO_2_ pressure, reaction temperature, reaction time, conversion/yield, selectivity, and recyclability, are compared where available. The summarized results provide an overview of the catalytic performance of different systems and help elucidate the characteristics and limitations of the reported catalysts.

## 3. Summary and Future Trends

This article provides a comprehensive review of the chemical fixation methods for synthesizing cyclic carbonates from CO_2_ and epoxides, with a detailed discussion on the application of various catalysts in this CO_2_ fixation process. Cyclic carbonates, as chemicals with a wide range of applications, are not only instrumental in reducing greenhouse gas emissions but also in converting CO_2_ into high-value-added products. The review focuses on catalyst systems such as alkali and alkaline earth metal salts, ionic liquids, metal salen complexes, hydrogen bond donors, MOFs, metal oxides, organic polymer supports, silica-based carriers, and carbon-based carriers. These catalysts play a crucial role in reducing the activation energy of the reaction, enhancing reaction efficiency, and improving selectivity. By continuously optimizing the catalyst structure and reaction conditions, the goal of converting CO_2_ into cyclic carbonates under mild conditions has been achieved, offering new perspectives and methodologies for the development of green and sustainable chemical processes.

Despite certain advancements in the field of CO_2_ chemical fixation and cyclic carbonate synthesis, challenges remain. From the perspective of practical scalability, the reported catalytic systems should be evaluated not only in terms of catalytic activity and selectivity but also with respect to catalyst loading, stability, recyclability, recovery, substrate scope, and product separation. Although most reported studies have been conducted at the laboratory scale, several systems have demonstrated characteristics that are favorable for further scale-up. For example, the 2-picolinic acid/*n*-Bu_4_NI system achieved yields of up to 97% and selectivities above 99% at relatively low catalyst loadings (1–2 mol%), and scale-up experiments further demonstrated its practical applicability. In addition, several heterogeneous catalysts based on silica and porous materials have shown good recyclability and facile recovery, which may reduce catalyst usage and facilitate downstream separation. For catalyst systems without direct scale-up experiments, their practical scalability can only be assessed preliminarily based on these experimentally demonstrated characteristics. Upon increasing the reaction scale, challenges associated with catalyst lifetime, heat and mass transfer, CO_2_ utilization efficiency, catalyst recovery, and product separation may become more pronounced. Therefore, further studies involving reactor optimization, long-term catalyst stability tests, process integration, and techno-economic evaluation are required to systematically establish the practical feasibility of these catalytic systems for industrial application.

From a reactor perspective, batch operation may remain useful for flexible substrate screening and processes involving relatively viscous or heterogeneous catalyst systems, whereas continuous-flow reactors may offer advantages in heat and mass transfer, CO_2_ utilization, process control, and catalyst productivity during scale-up. However, continuous operation also requires effective catalyst retention and resistance to deactivation. When moving beyond neat epoxide feeds toward more realistic feed compositions, catalyst leaching and deactivation may become more significant, while catalyst separation may become more challenging, potentially affecting catalyst lifetime and process efficiency. Therefore, the development of robust catalysts with long-term stability, facile recovery, and compatibility with realistic feed compositions is essential for practical scale-up and continuous-flow processes.

Looking ahead, future research should extend beyond improving catalytic activity and focus on the development of more sustainable and economically viable catalytic processes. In particular, the design of catalysts with reduced environmental impact, together with the utilization of biomass and industrial waste as raw materials or catalyst precursors, may promote resource recycling and reduce dependence on fossil resources. Further efforts should also explore photocatalytic and electrocatalytic approaches for CO_2_ conversion, particularly those driven by renewable energy, to provide alternative pathways for the chemical transformation of CO_2_. At the process level, integrating catalyst design with reactor engineering, catalyst recovery, product separation, and process optimization will be important for translating laboratory-scale results into practical applications. By addressing these aspects in an integrated manner, future studies may facilitate the development of more efficient, economical, and sustainable CO_2_ conversion processes.

## Figures and Tables

**Figure 1:**
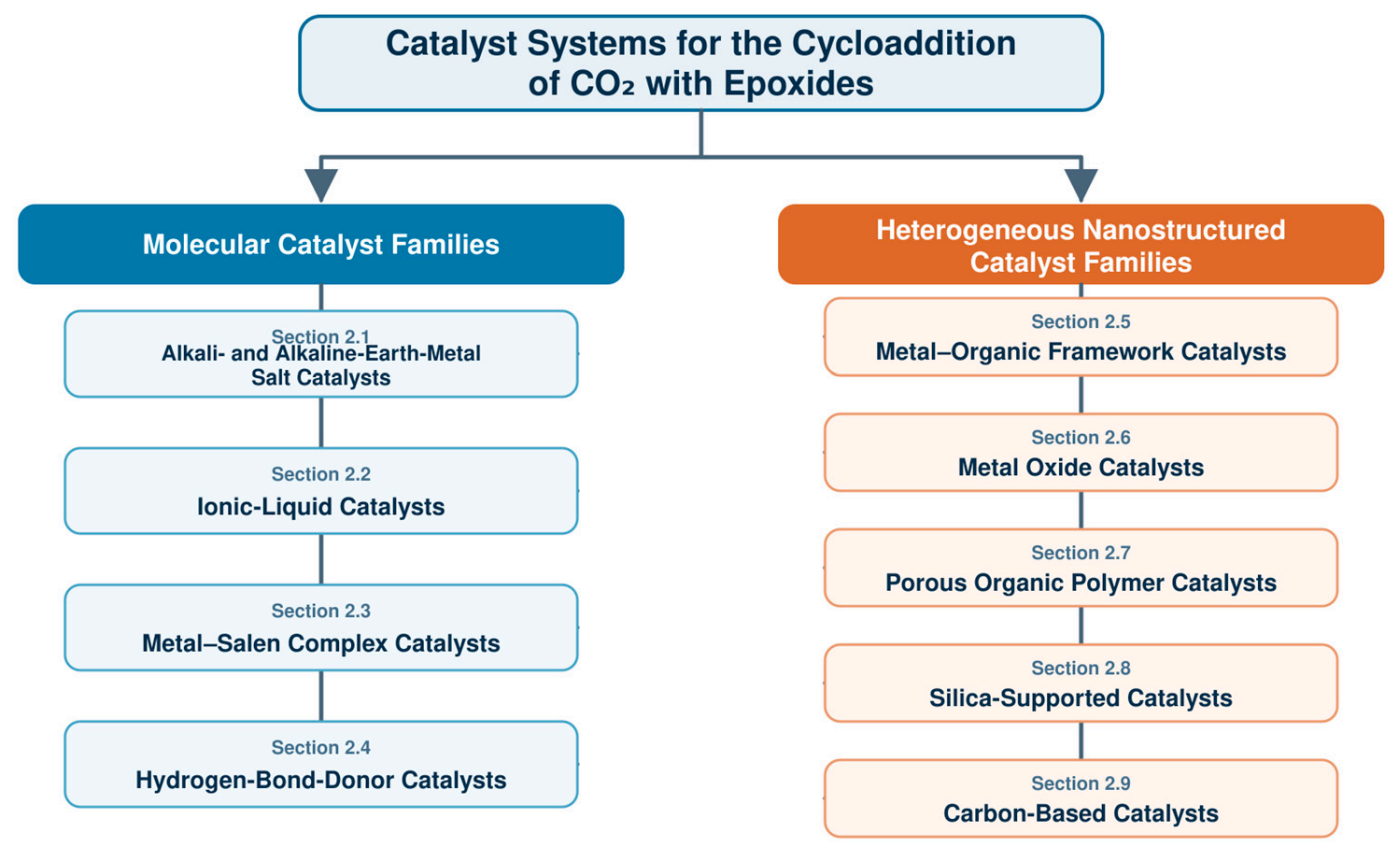
Classification of catalyst systems for the cycloaddition of CO_2_ with epoxides into molecular catalyst families and heterogeneous nanostructured catalyst families.

**Figure 2:**
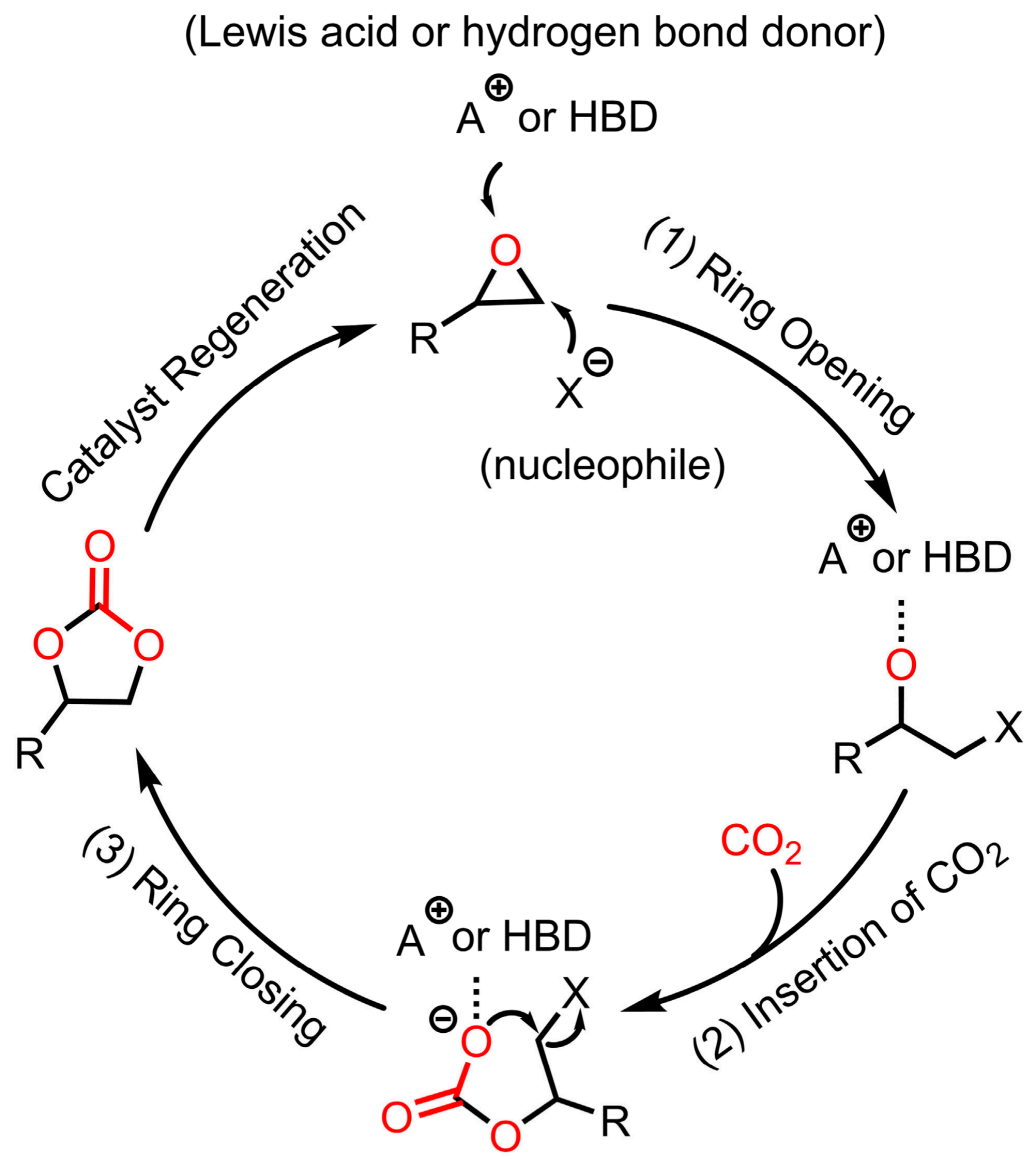
General mechanism for the cyclization of CO_2_ and epoxide to form cyclic carbonate.

**Figure 3:**
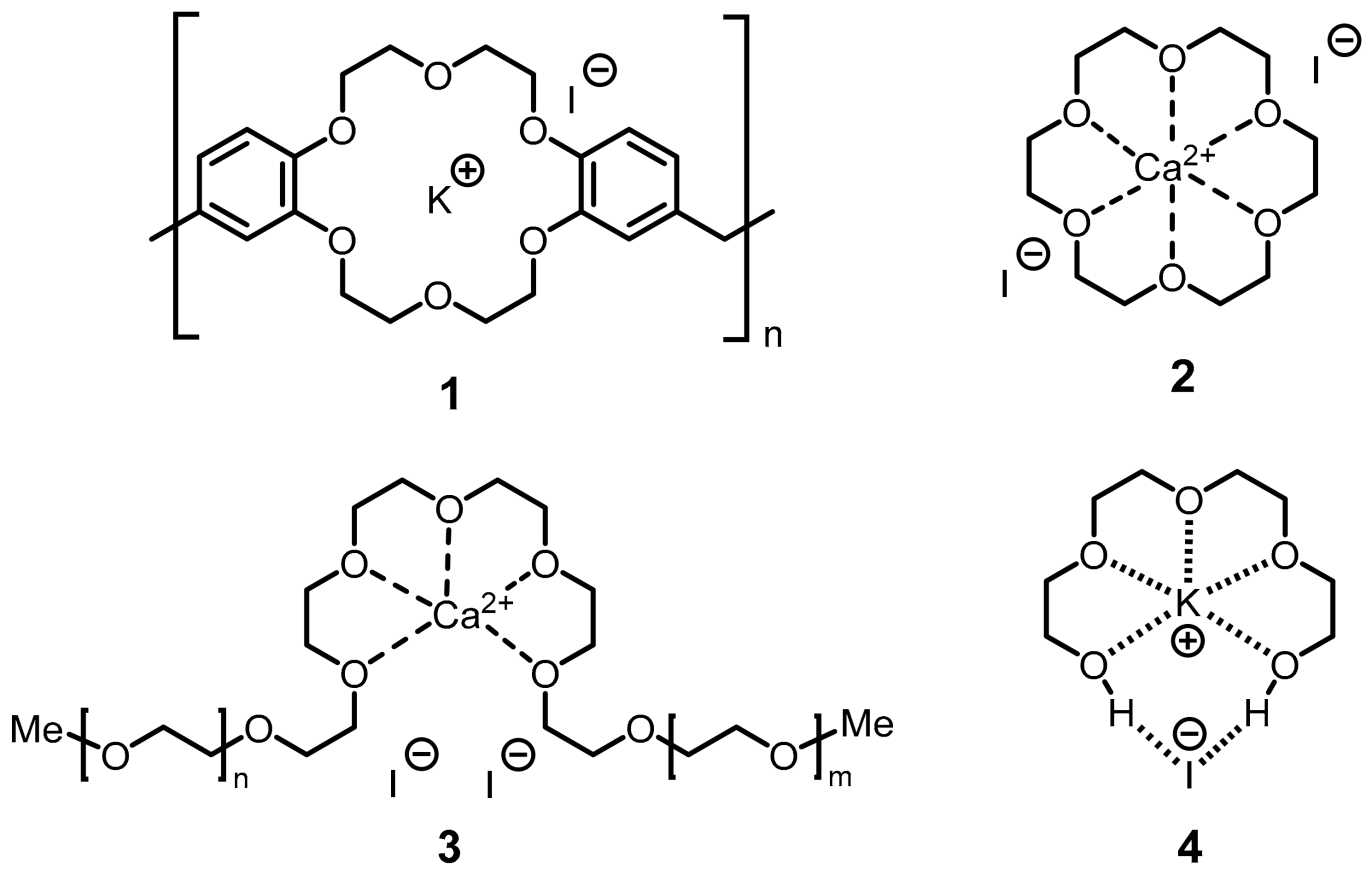
Chemical structures of catalysts **1**–**4** discussed in this section.

**Figure 4:**
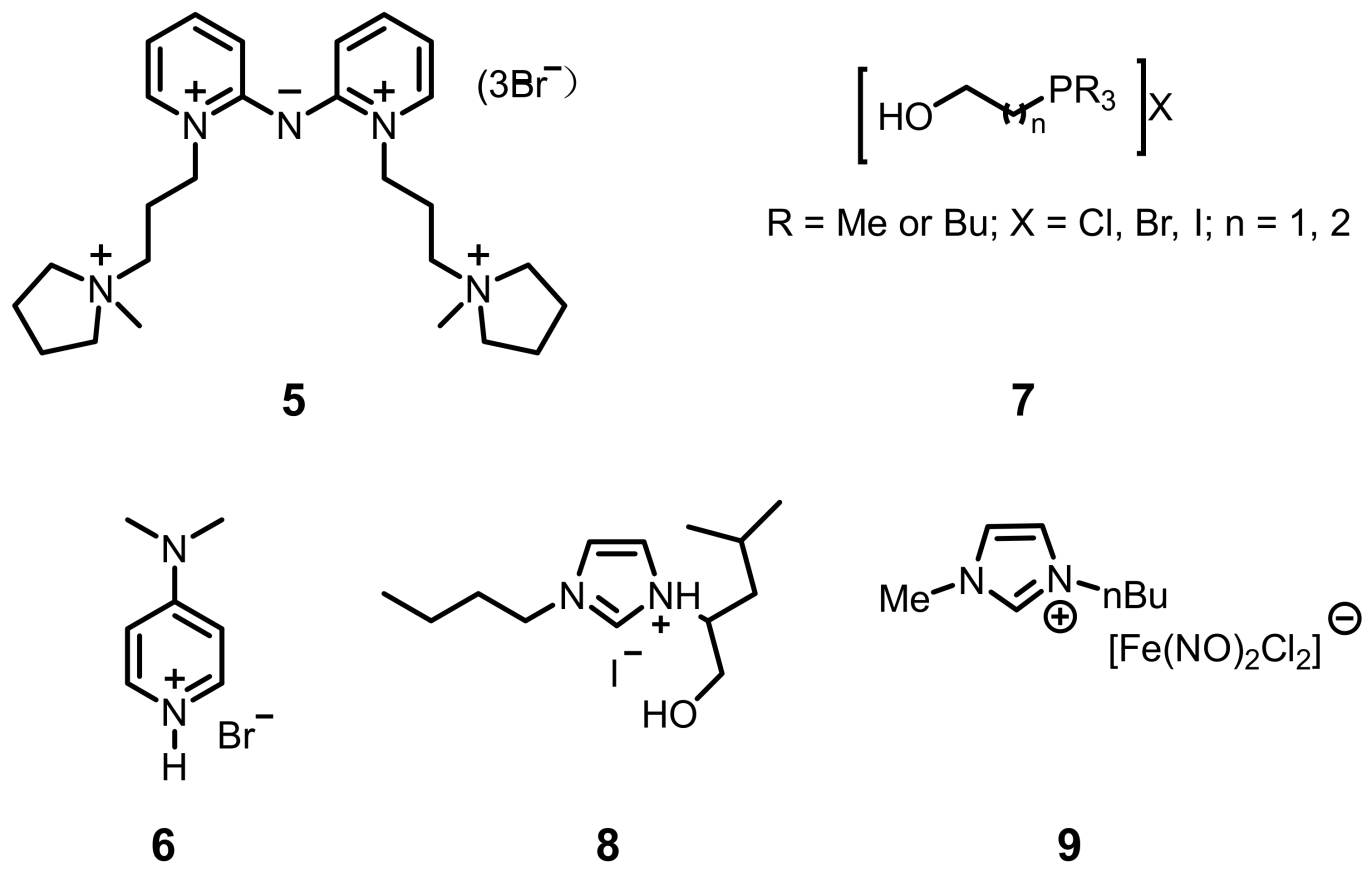
Chemical structures of ionic-liquid catalysts **5**–**9** used for the cycloaddition of CO_2_ with epoxides. Catalysts **5** and **6** are pyridinium-based organocatalysts, whereas **7** and **8** contain hydroxyl groups that promote epoxide activation through hydrogen-bonding interactions. In the iron-containing imidazolium system **9**, the iron species acts as a Lewis acid for epoxide activation, while the chloride ion serves as the nucleophile for ring opening.

**Figure 5:**
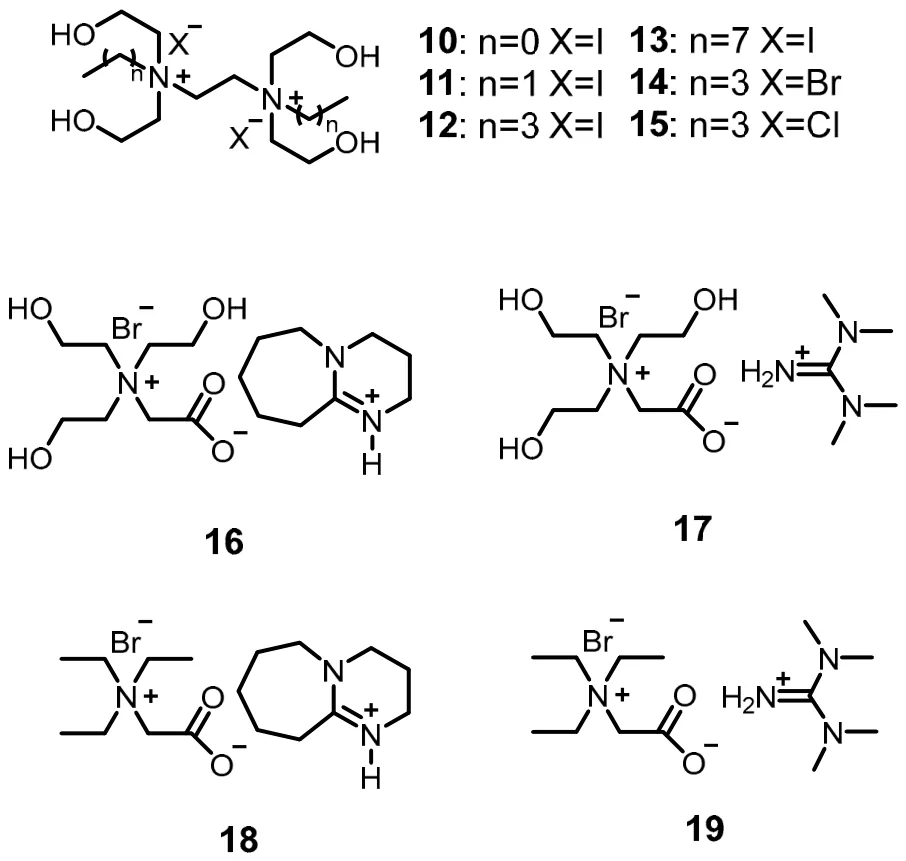
Chemical structures of multihydroxyl bis(quaternary ammonium) ionic liquids **10**–**15** and diionic ammonium salts **16**–**19** used as metal-free catalysts for the cycloaddition of CO_2_ with epoxides. Compounds **10**–**15** differ in spacer length and halide counterion, whereas the catalytic behavior of **16**–**19** is associated with the cooperative action of their cationic and anionic sites.

**Figure 6:**
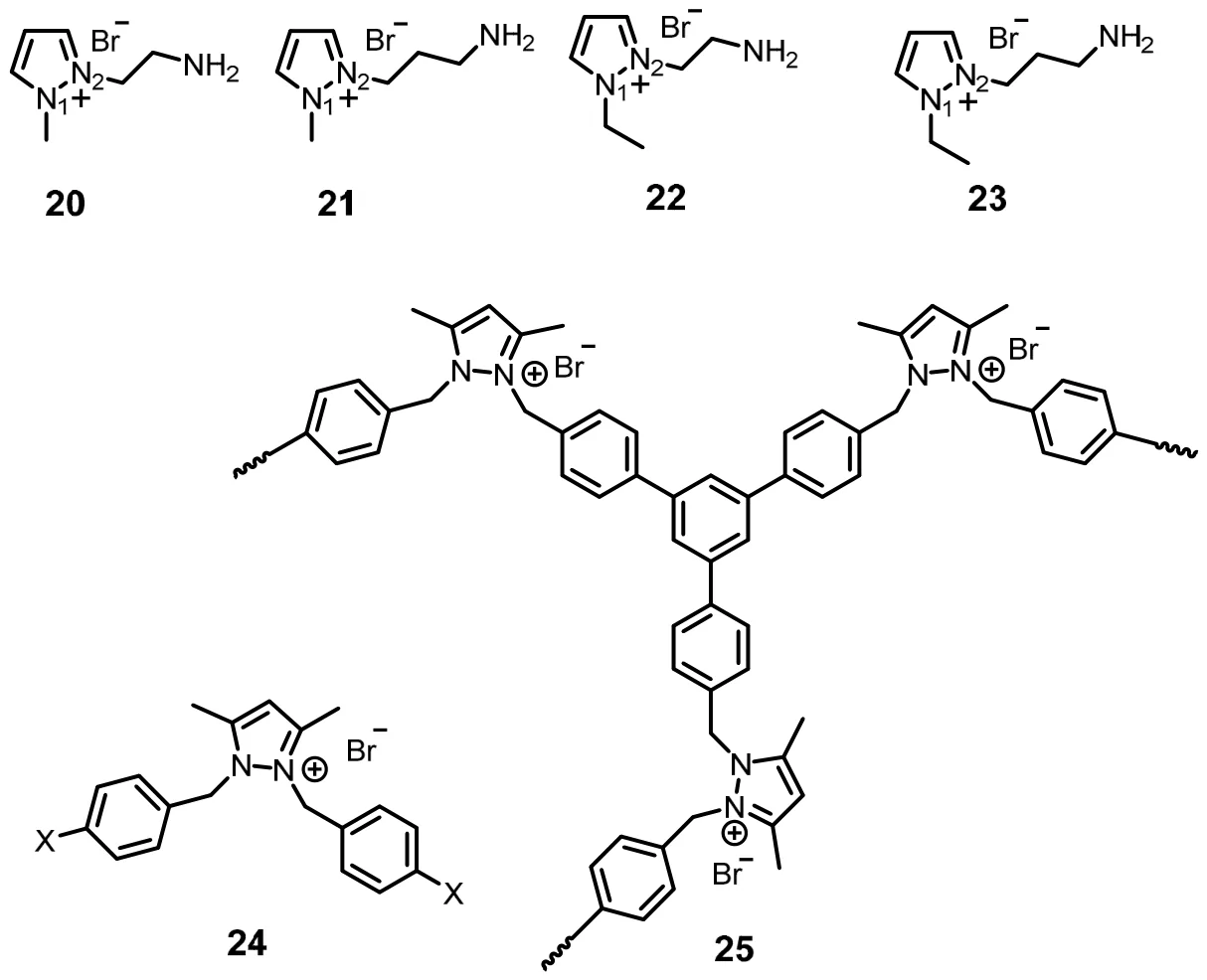
Chemical structures of amino-functionalized pyrazolium ionic liquids **20**–**23**, the pyrazole-based ionic liquid **24**, and its corresponding poly(ionic liquid) **25** used for the metal-free cycloaddition of CO_2_ with epoxides. Compounds **20**–**24** represent molecular ionic catalysts, whereas **25** incorporates related ionic catalytic units into a polymeric architecture.

**Figure 7:**
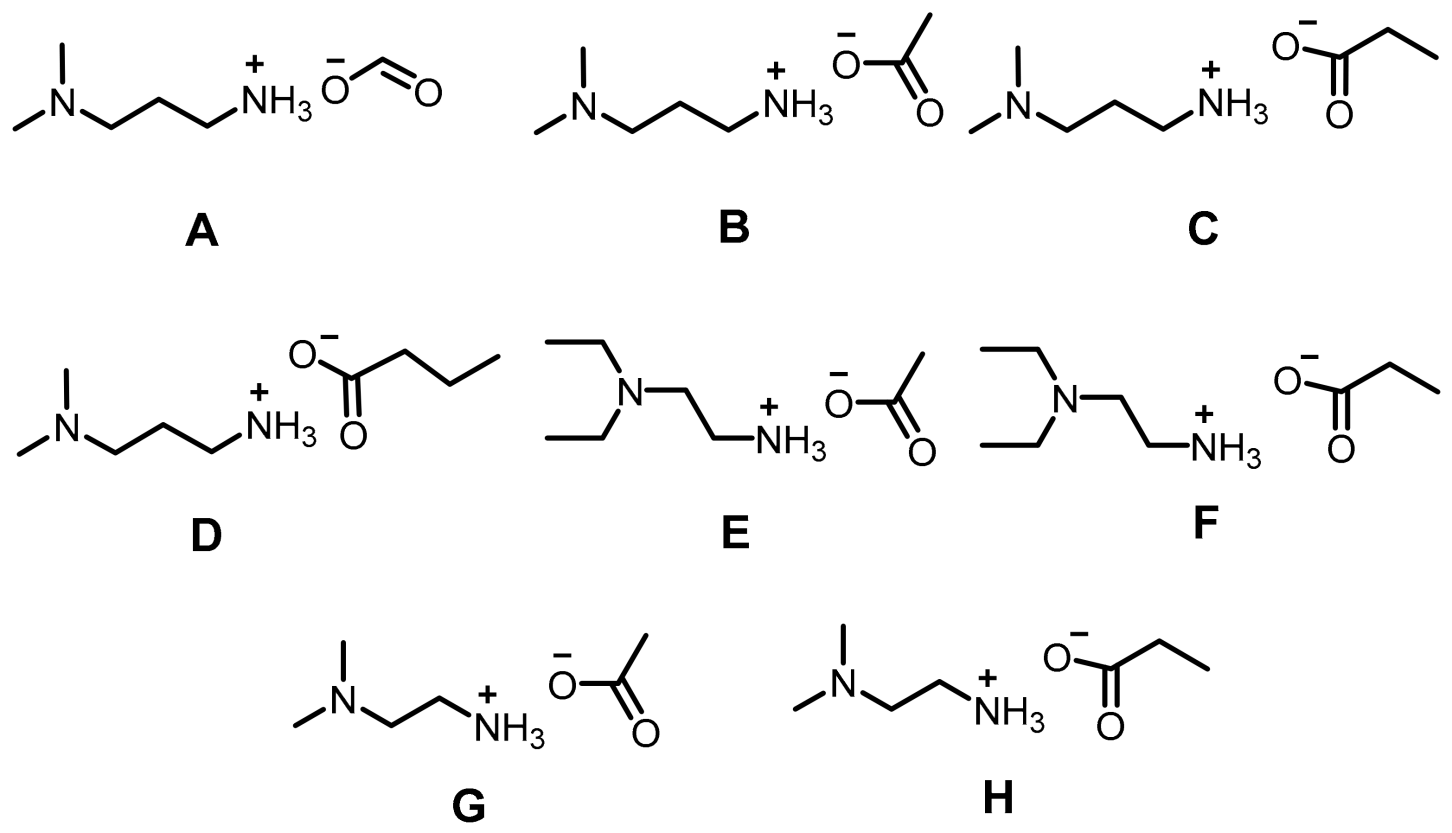
Chemical structures of protic ionic liquids (PILs) (**A**–**H**) prepared from low-cost amines and carboxylic acids for integrated CO_2_ capture and catalytic cycloaddition with epoxides under metal- and solvent-free conditions.

**Figure 8:**
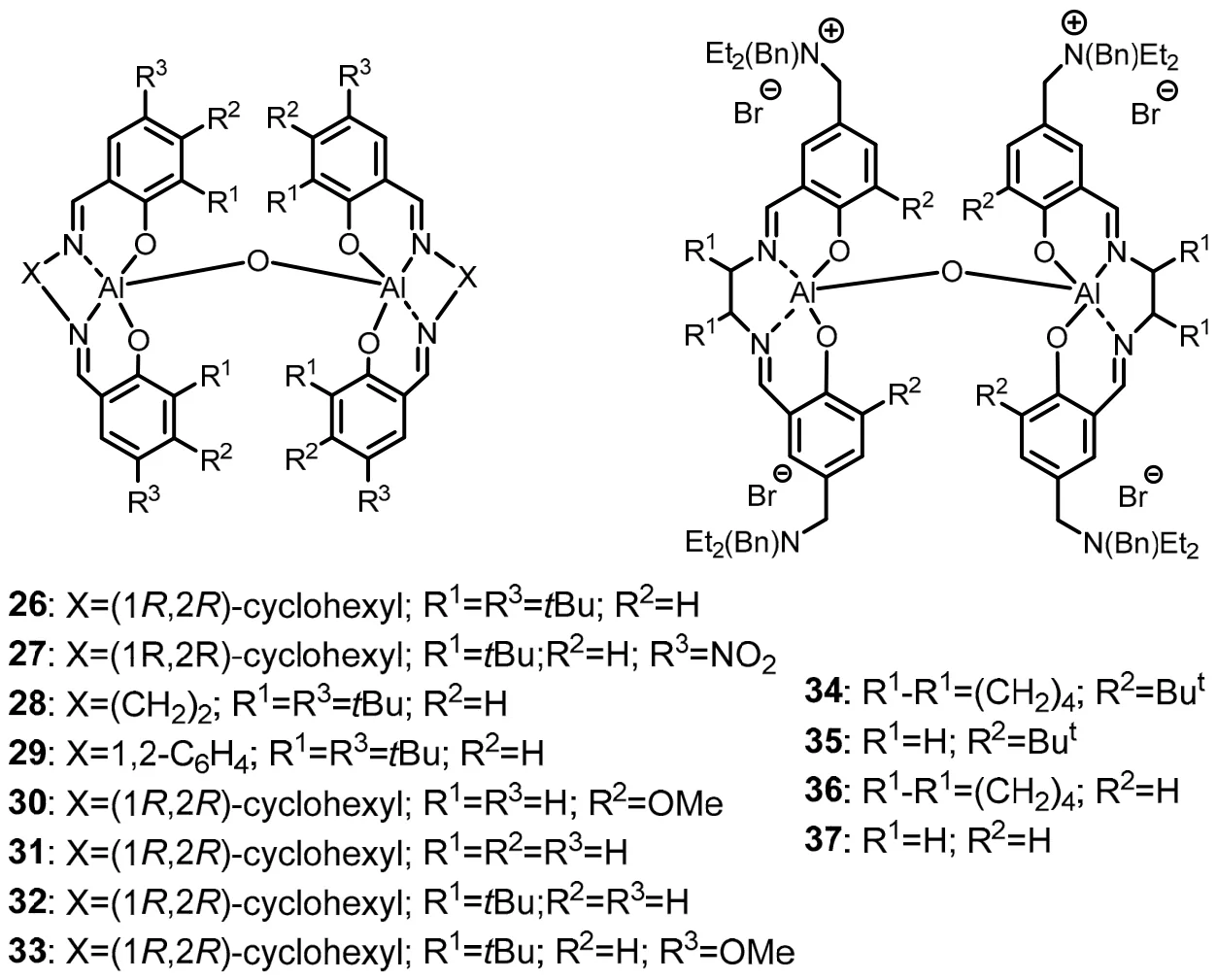
Chemical structures of bimetallic aluminum(salen) complexes **26**–**33** and single-com ponent bifunctional aluminum(salen) catalysts **34**–**37** for the cycloaddition of CO_2_ with epoxides. Complexes **26**–**33** are used with tetrabutylammonium bromide (TBAB) as an external co-catalyst, whereas **34**–**37** contain covalently tethered tetraalkylammonium bromide groups, integrating the metal centers and nucleophilic bromide ions within the same catalyst.

**Figure 9:**
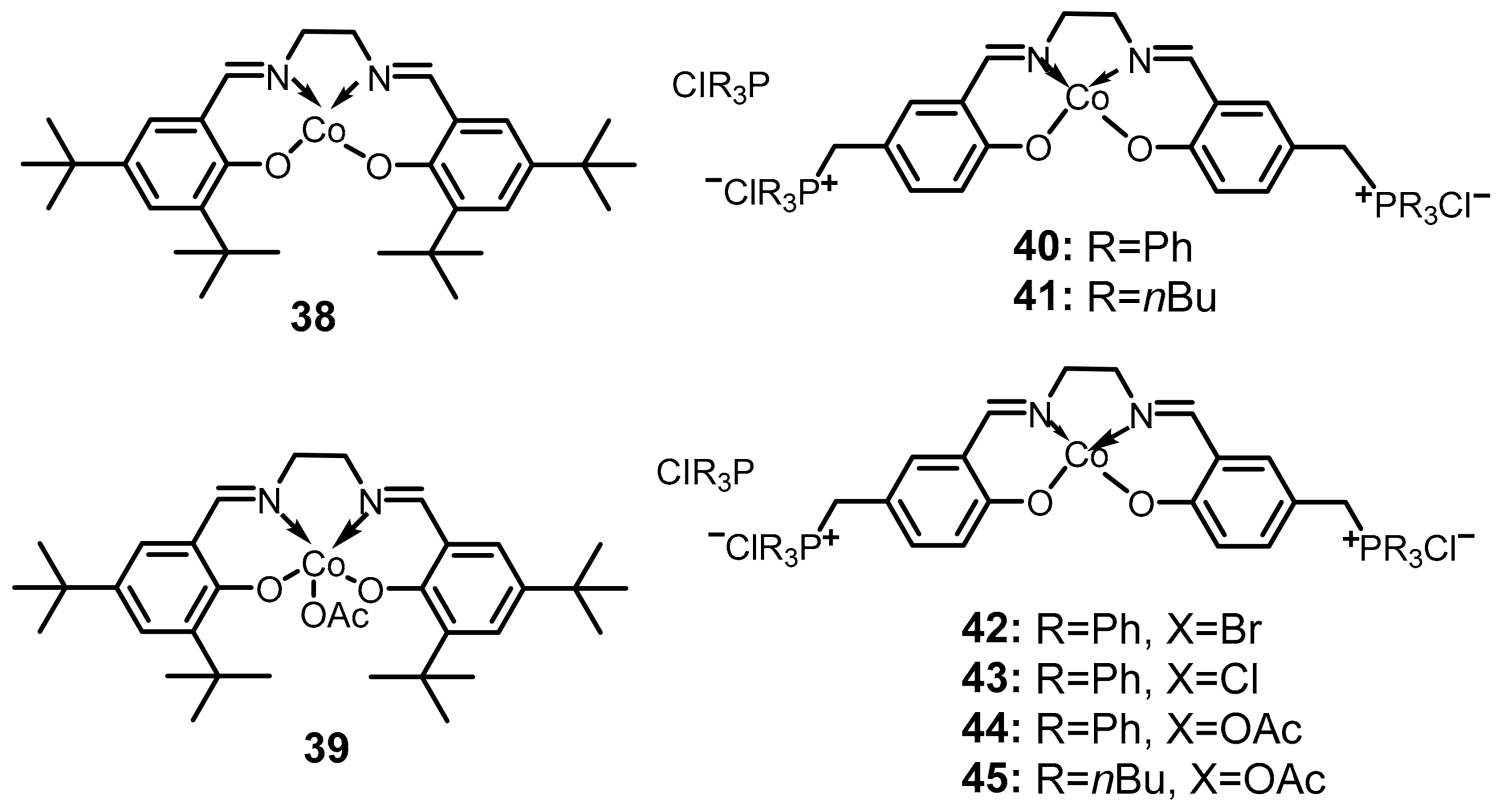
Chemical structures of cobalt(salen) complexes **38**–**45** used for the cycloaddition of CO_2_ with epoxides. Complexes **40**–**45** incorporate pendant quaternary phosphonium groups, combining Lewis acidic Co centers with proximal ionic components, while **42**–**45** contain different axial ligands (X = Br, Cl, or OAc).

**Figure 10:**
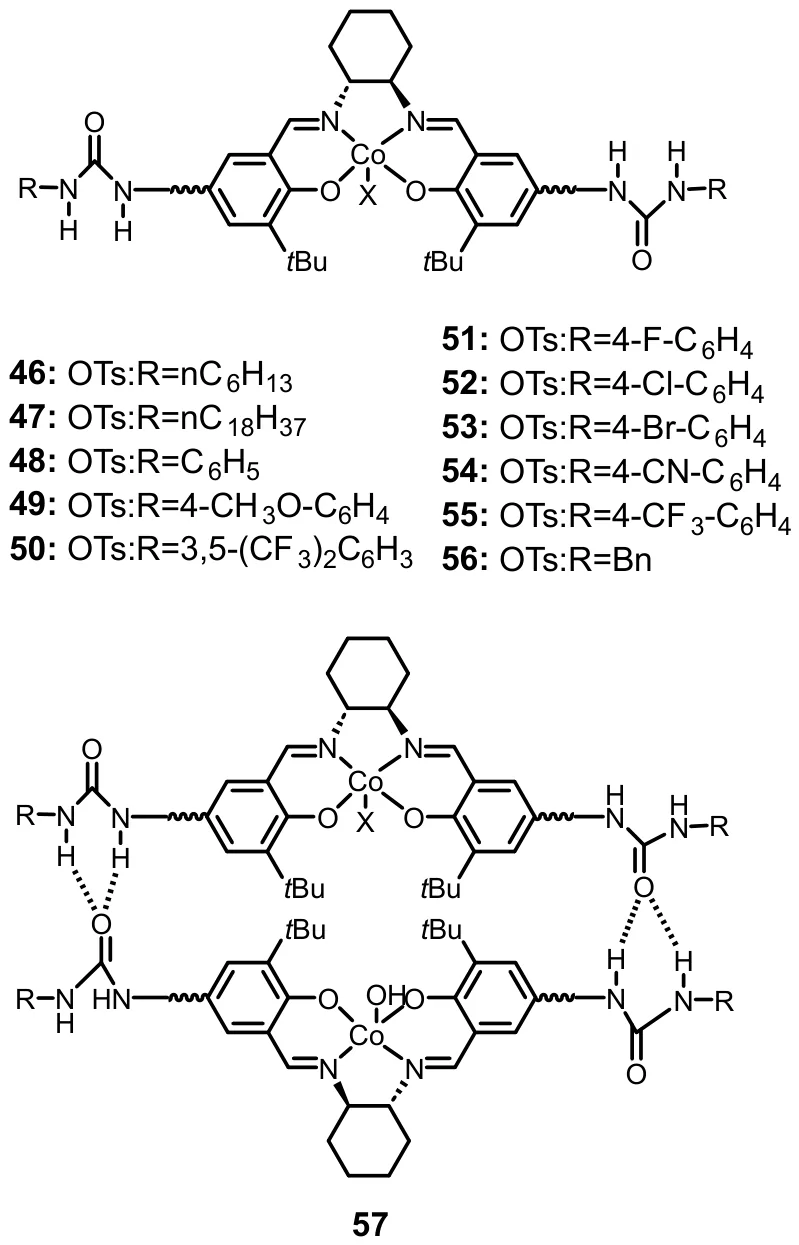
Chemical structures of bis-urea-functionalized cobalt(salen) complexes **46**–**56** and their hydrogen-bonded self-assembled dimer **57**. Intermolecular urea–urea hydrogen bonds bring two cobalt(salen) units into proximity, enabling cooperative activation and thereby enhancing catalytic activity in the cycloaddition of CO_2_ with epoxides.

**Figure 11:**
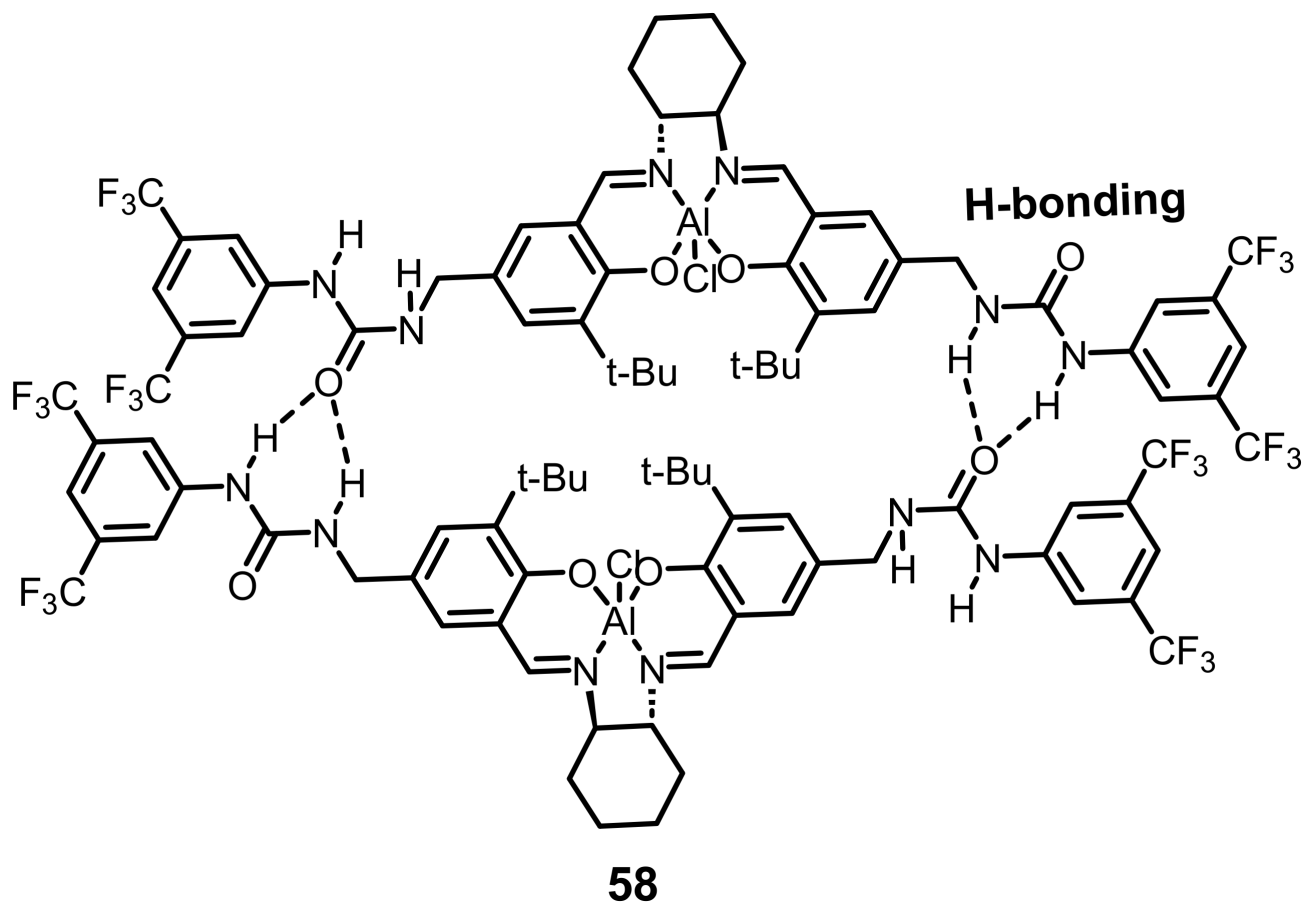
Hydrogen-bond-mediated self-assembly of the bimetallic aluminum(salen) catalyst **58**. Intermolecular urea–urea hydrogen bonds bring two aluminum(salen) units into proximity, generating a cooperative bimetallic environment for the cycloaddition of CO_2_ with epoxides [58].

**Figure 12:**
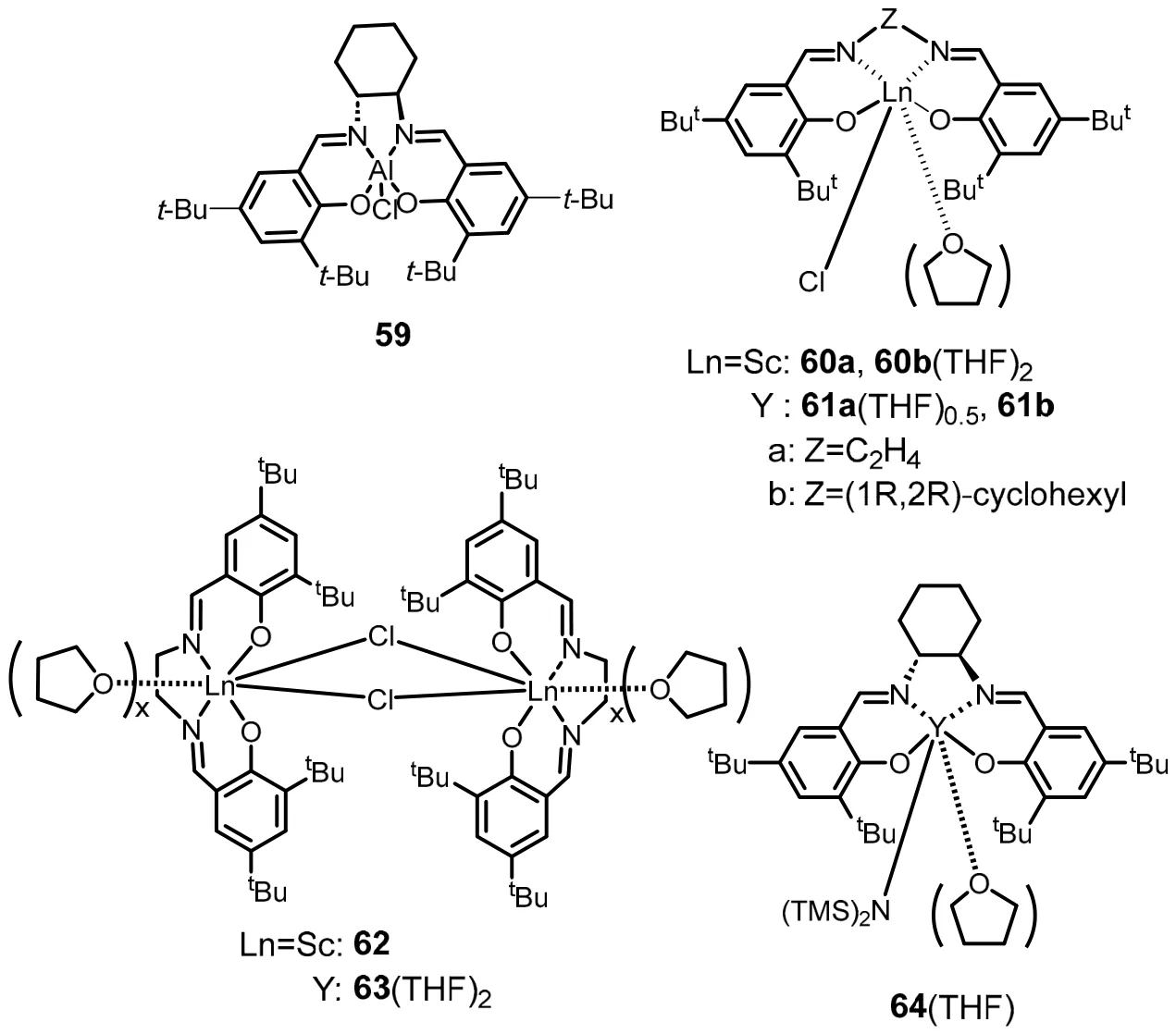
Chemical structures of the monomeric aluminum(salen) complex **59** and the mono- and binuclear Sc/Y-based rare-earth salen complexes **60**–**64**. Complexes **60a**,**b**, **61a**,**b**, and **64** are mononuclear, whereas **62** and **63** contain chloride-bridged dinuclear metal centers. DFT calculations attributed the catalytic behavior of the Sc/Y complexes to their relatively low reaction barriers and reduced steric hindrance.

**Figure 13:**
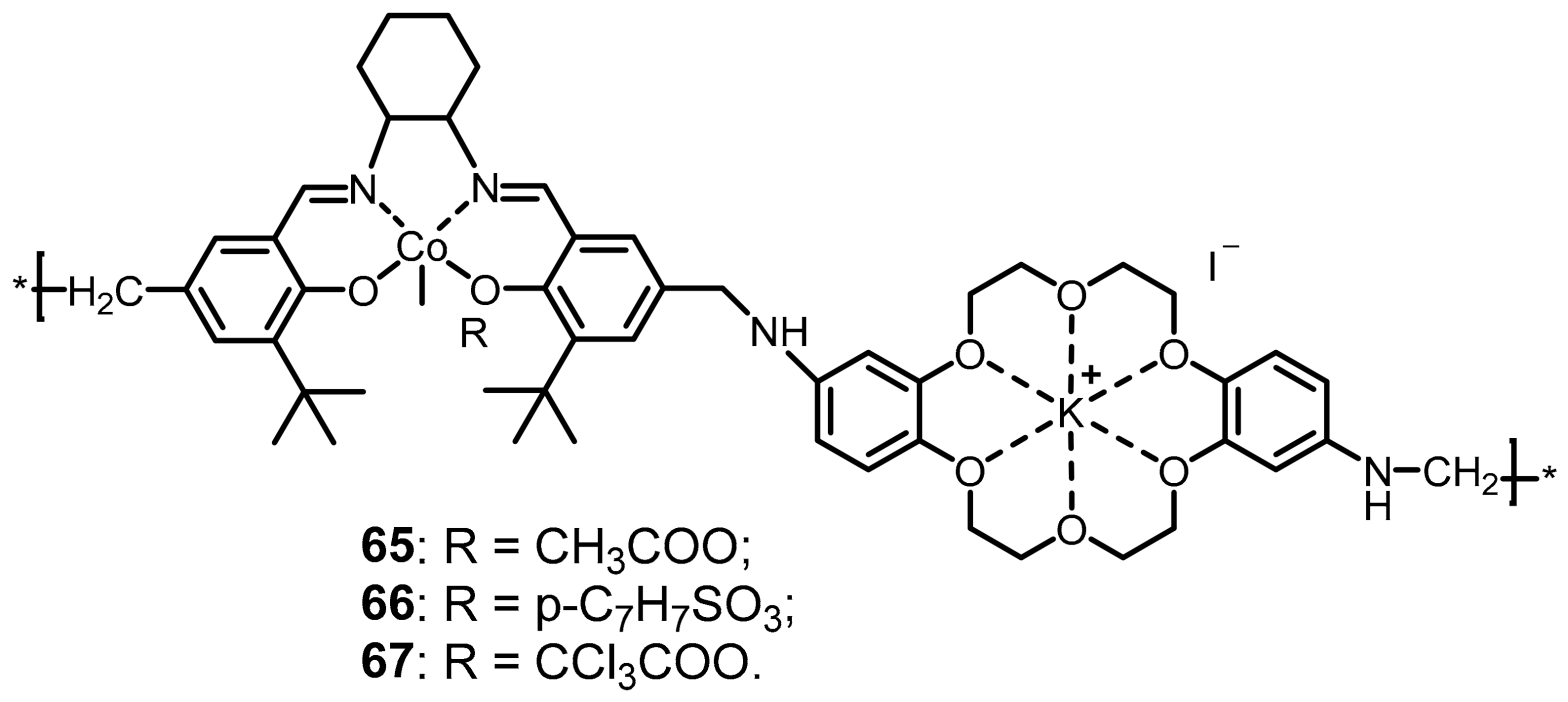
Chemical structures of cobalt–salen porous organic catalysts **65**–**67** containing different axial anions and crown-ether-coordinated KI sites. * denotes the porous organic catalyst support.

**Figure 14:**
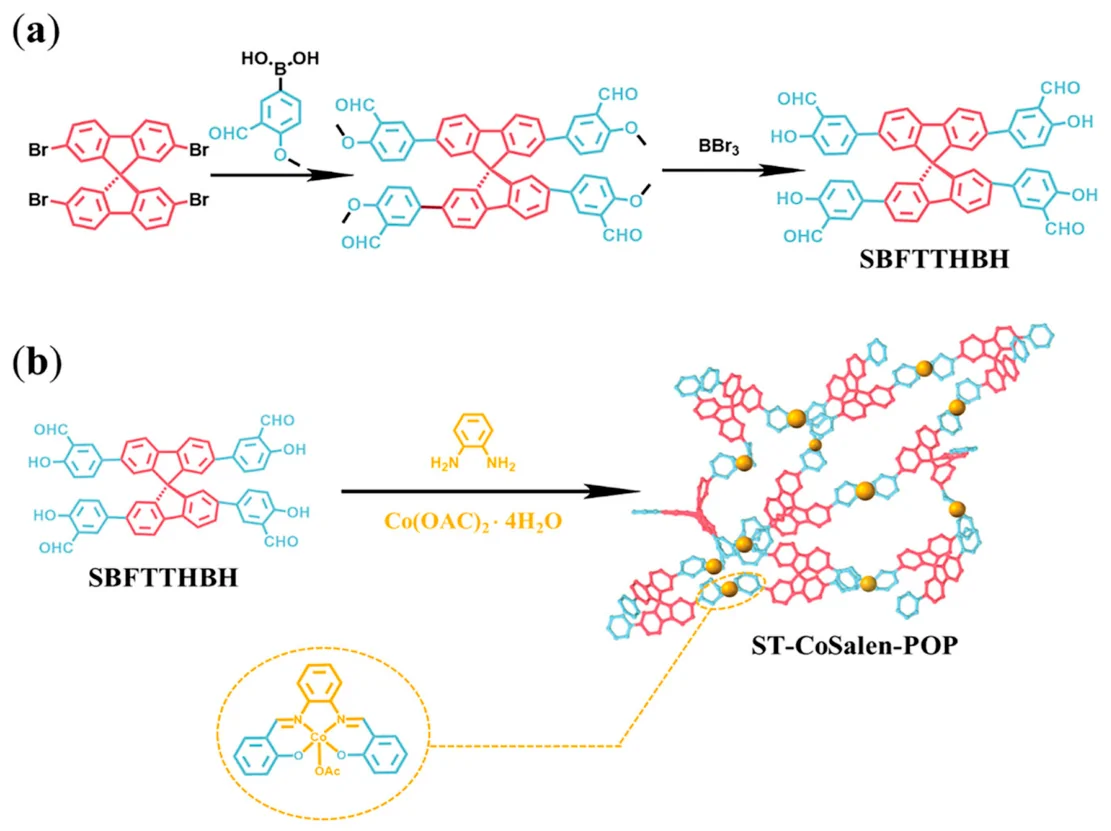
Synthesis of ST-CoSalen-POP: (**a**) preparation of SBFTTHBH by coupling and BBr_3_-mediated demethylation; (**b**) condensation with o-phenylenediamine followed by coordination with Co(OAc)_2_·4H_2_O. The Co–salen centers provide Lewis acidic sites, while the porous spirobifluorene framework immobilizes and disperses these catalytic sites. Reproduced from Ref. [61] with permission.

**Figure 15:**
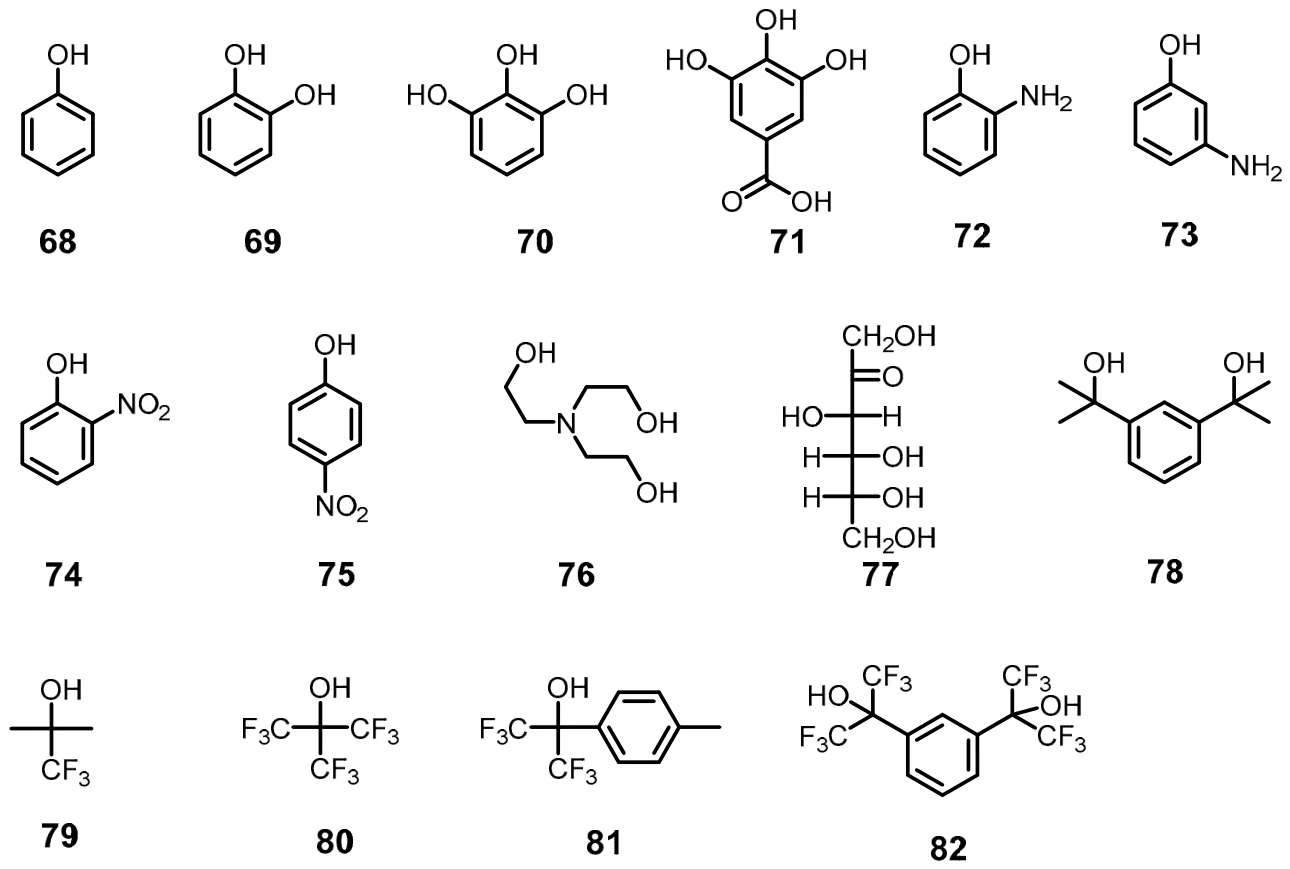
Chemical structures of organic hydrogen-bond donors **68**–**82** evaluated with tetrabutylammonium bromide (TBAB) for CO_2_–epoxide cycloaddition.

**Figure 16:**
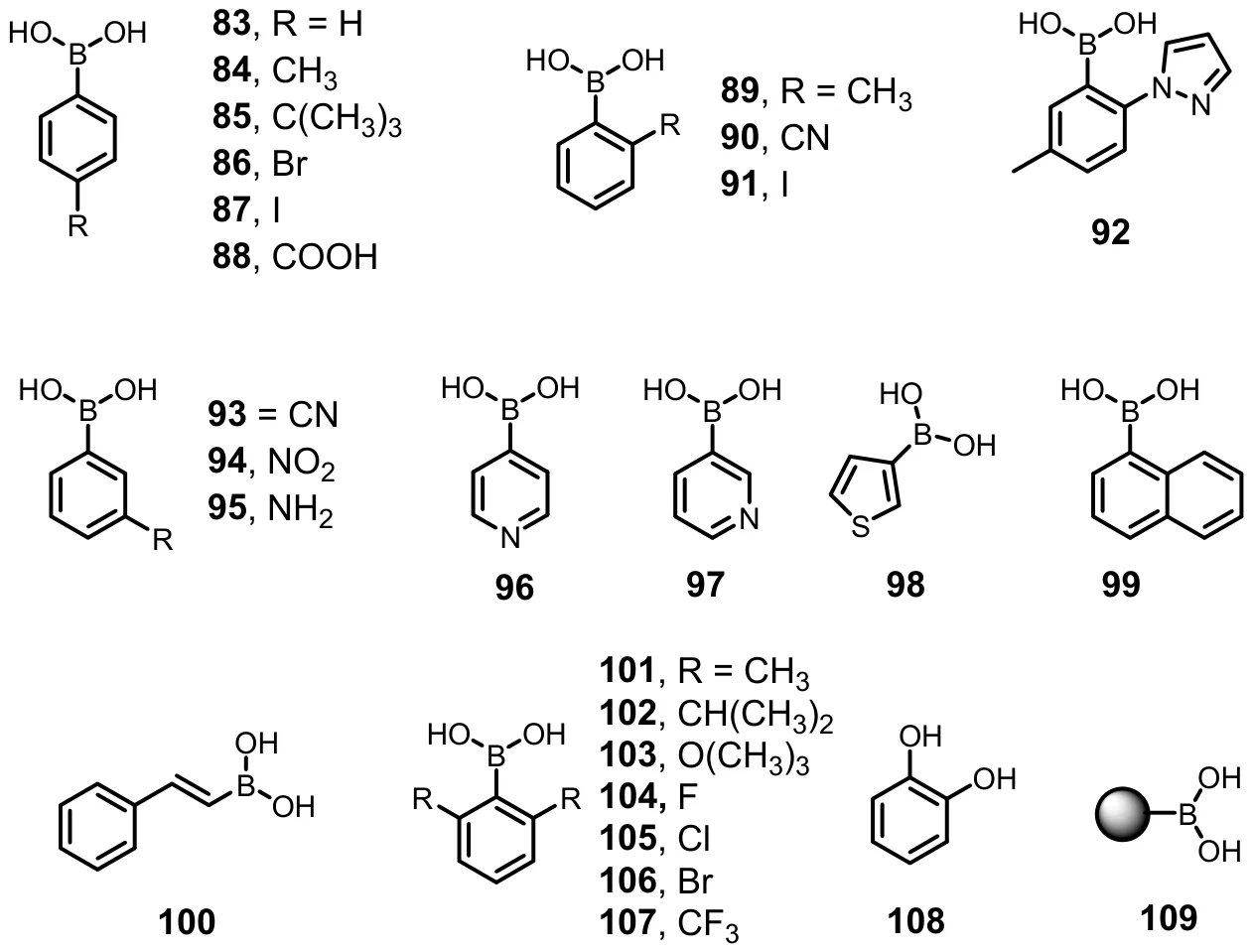
Chemical structures of boronic-acid-based hydrogen-bond donors **83**–**109** evaluated with TBAI for the cycloaddition of CO_2_ with epoxides.

**Figure 17:**
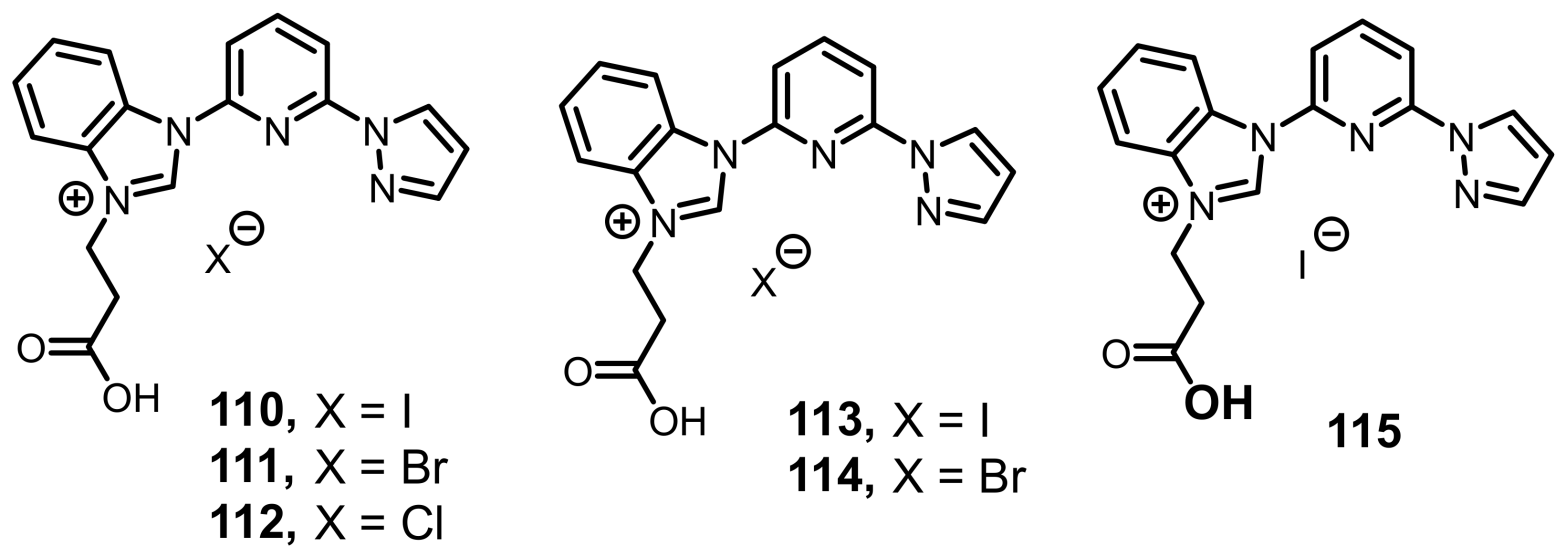
Chemical structures of pyridine-bridged benzimidazolium salts **110**–**115** used as N-heterocyclic carbene precursors in the CrCl_2_/TBAB catalytic system for CO_2_–epoxide cycloaddition.

**Figure 18:**
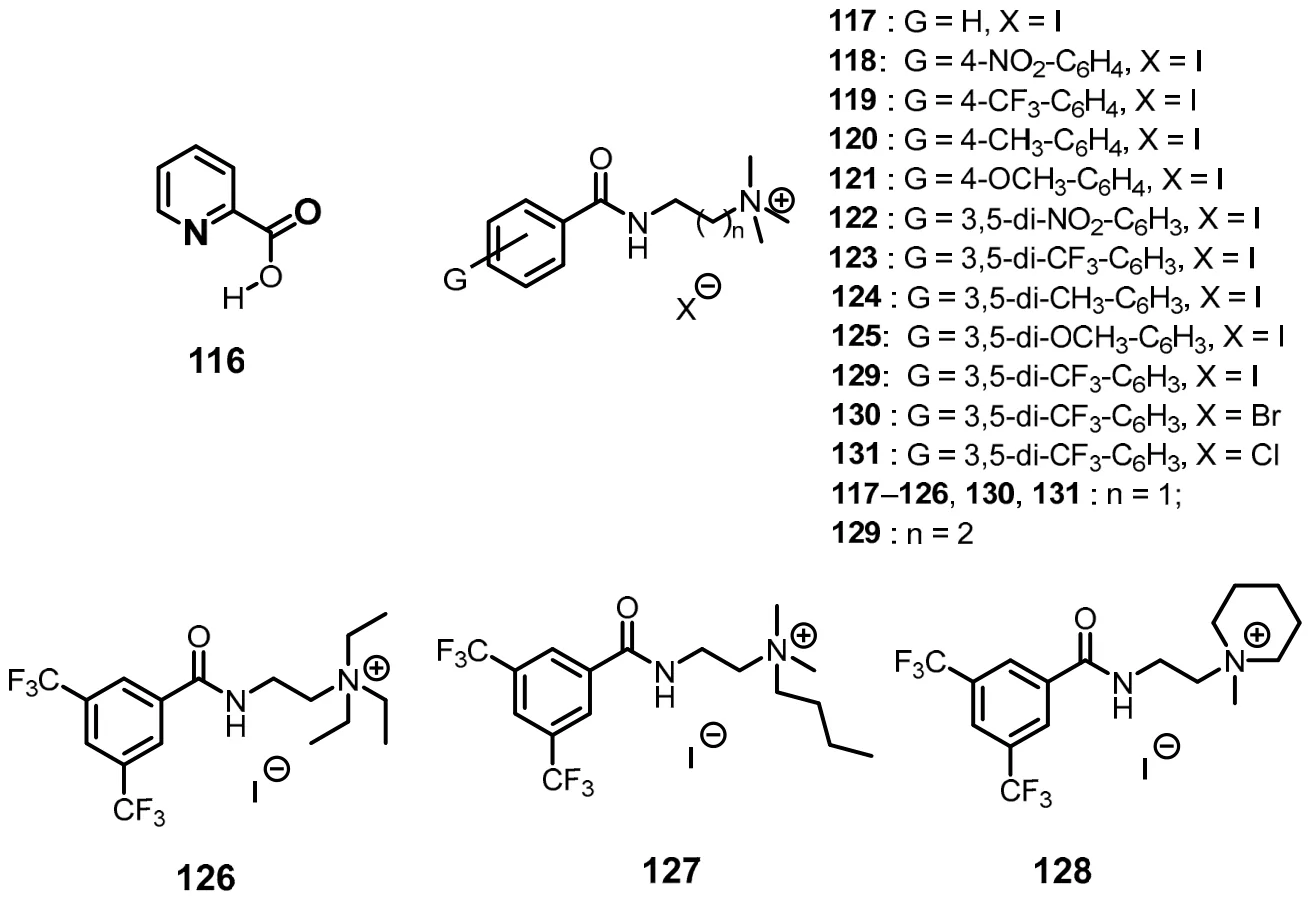
Chemical structures of 2-picolinic acid **116** and bifunctional amide-containing quaternary ammonium catalysts **117**–**131**. Compound **116** is used with n-Bu_4_NI, whereas **117**–**131** combine a hydrogen-bond-donating amide group with a nucleophilic halide anion.

**Figure 19:**
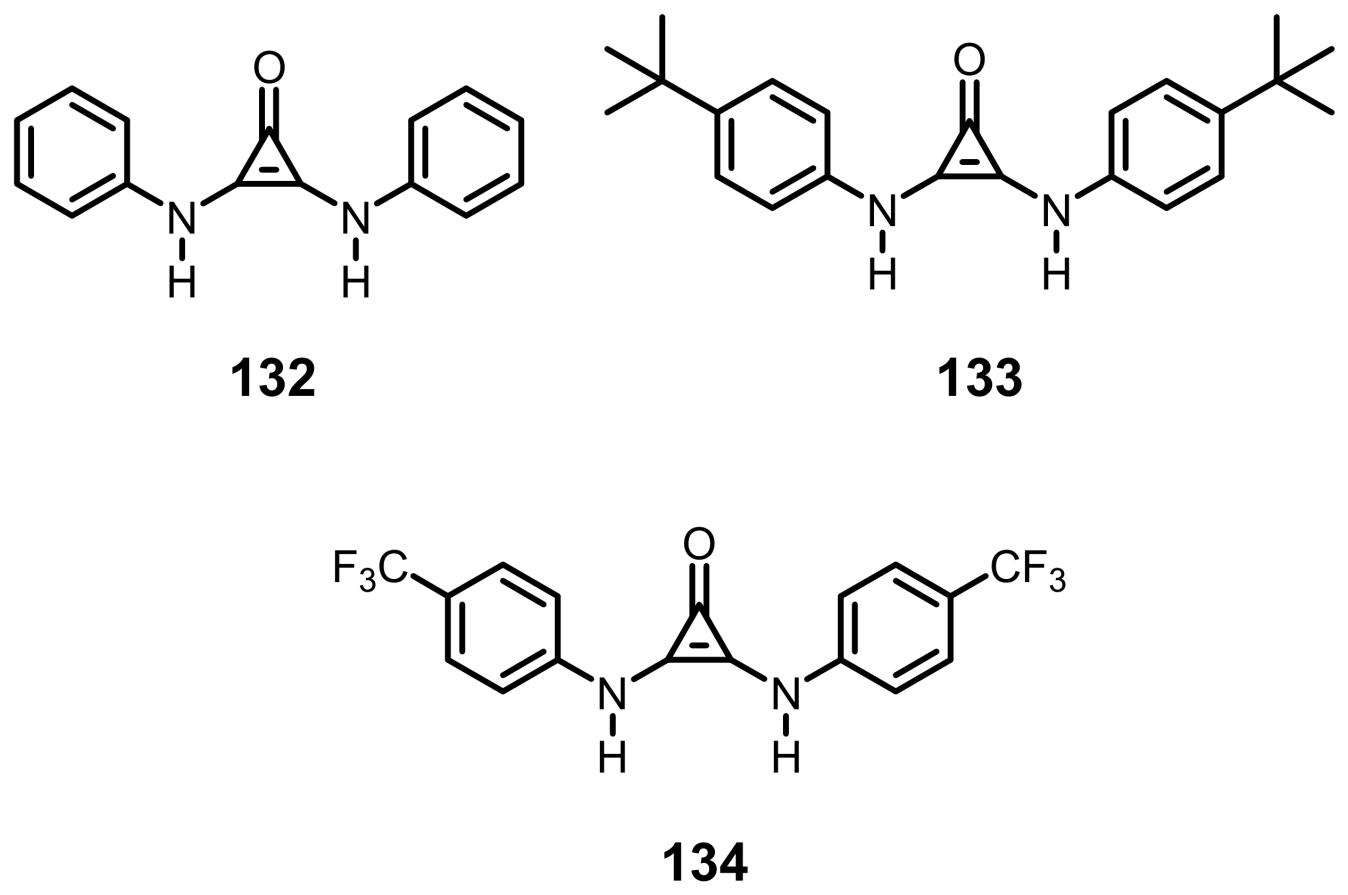
Chemical structures of deltamide-based bidentate hydrogen-bond-donor catalysts **132**–**134**.

**Table 1:** Comparison of representative catalysts for the synthesis of cyclic carbonates from CO_2_ and epoxides.

Catalyst Type	Representative Catalyst	Substrate	T (°C)	CO_2_ Pressure	Reaction Time	Yield/Conversion	Selectivity	Recyclability	Ref.
Alkali/alkaline-earth metal salts	NaBr-supported liquid-phase catalyst	Propylene oxide	150	5.5 MPa	6 h	96.39% yield	No apparent by-products observed	4 cycles	[31]
Alkali/alkaline-earth metal salts	NaI·2H_2_O	Propylene oxide	120	1.0 MPa	1.5 h	97% yield	~99%	NR	[32]
Alkali/alkaline-earth metal salts	MgBr_2_/TPP	Butadiene monoxide	RT	3.5 MPa	8 h	95.2% yield	No apparent by-products observed	6 cycles	[34]
Alkali/alkaline-earth metal salts	KI@poly18C6	1,2-Epoxybutane	80	1.0 MPa	14 h	93% yield	NR	23 cycles (with regeneration)	[36]
Ionic liquids	[DMAPH]Br	Styrene oxide	120	0.1 MPa	4 h	96% conversion	99%	5 cycles without significant activity loss	[45]
Ionic liquids	Multihydroxyl bis(quaternary ammonium) IL	Propylene oxide	120	2.0 MPa	3 h	99.4% yield	>99%	≥5 cycles	[49]
Ionic liquids	Pyrazole-based poly(ionic liquid) (DDP)	Epichlorohydrin	120	0.1 MPa	7 h	99% yield	99%	10 cycles without significant activity loss	[52]
Metal salen complexes	K@SC-3 (Co–salen/crown ether porous catalyst)	Epichlorohydrin	90	0.1 MPa	24 h	96.5% yield	NR	6 cycles with slight activity loss	[60]
Metal salen complexes	ST-CoSalen-POP/TBAB	Propylene oxide	25	0.1 MPa	36 h	96% yield	NR	3 cycles with stable activity	[61]
Hydrogen-bond donors	2-Picolinic acid/n-Bu_4_NI	Butylene oxide	50	0.1 MPa	18 h	97% isolated yield	≥99%	5 cycles (92% conversion in 5th run)	[67]
Hydrogen-bond donors	DT3/ NBu_4_I	Styrene oxide	100	0.1 MPa	12 h	95% conversion	99%	NR	[69]
MOFs	Cu-URJC-8/TBAB	Epichlorohydrin	RT	1.2 MPa	24 h	90% conversion (89% yield)	>99%	NR	[75]
MOFs	Zn-URJC-12/TBAB	Epichlorohydrin	RT	1.2 MPa	24 h	90% conversion	>99%	5 cycles (conversion from 90% to 83%)	[76]
Metal oxides	0.75 wt% Li-MgO	Epichlorohydrin	130	3.0 MPa	4 h	98.2% conversion (96% isolated yield)	~100%	3 cycles without significant activity loss	[78]
Metal oxides	Mesoporous MgO/TBAB	Epichlorohydrin	RT	0.1 MPa	4 h	99% yield (~99% conversion)	~100%	5 cycles without significant activity loss	[79]
Metal oxides	Cozi-nmf/TABA (CuO-ZnO)	Phenyl glycidyl ether	RT	0.1 MPa	3 h	97% isolated yield	NR	5 cycles (yield from 97% to90%)	[82]
Organic polymer supports	Mg–Por\DVB(1:20)@POPs/TBAB	Propylene oxide	120	3.0 MPa	1 h	58% yield	>99%	5 cycles without significant activity loss	[88]
Organic polymer supports	Cationic ionene D^1^,^4^ (Cl^−^)	Styrene oxide	120	0.1 MPa	8 h	>90% conversion	NR	≥4 cycles	[89]
Silica-supported catalysts	Silica-supported triethanolammonium iodide (4a)	1,2-Epoxybutane	90	1.0 MPa	6 h	99% isolated yield	≥98%	13 cycles (≥93% yield per cycle)	[97]
Silica-supported catalysts	Im-Si-I-5	Propylene oxide	110	2.5 MPa	4 h	96% yield	99%	4 cycles (91% yield in 4th reuse)	[98]
Silica-supported catalysts	Ag/TUD-1/TBAB	Epichlorohydrin	60	1.0 MPa	4 h	98% yield	NR	4 cycles with slight yield loss	[102]
Carbon-based catalysts	NSC-773/TBAI	Epichlorohydrin	70	0.1 MPa	24 h	95% conversion (88% yield)	98%	5 cycles with slight activity loss	[108]
Carbon-based catalysts	BPO_4_@BCN-800/KI	Epichlorohydrin	80	0.1 MPa	36 h	99% yield	99%	5 cycles with slight activity loss	[110]

Notes: RT = room temperature; NR = not explicitly reported.

## Data Availability

No new data were created or analyzed in this study. Data sharing is not applicable to this article.
